# Transcriptomic analysis reveals tomato genes whose expression is induced specifically during effector-triggered immunity and identifies the Epk1 protein kinase which is required for the host response to three bacterial effector proteins

**DOI:** 10.1186/s13059-014-0492-1

**Published:** 2014-10-17

**Authors:** Marina A Pombo, Yi Zheng, Noe Fernandez-Pozo, Diane M Dunham, Zhangjun Fei, Gregory B Martin

**Affiliations:** Boyce Thompson Institute for Plant Research, 533 Tower Road, Ithaca, NY 14853-1801 USA; Section of Plant Pathology and Plant-Microbe Biology, School of Integrative Plant Science, Cornell University, Ithaca, NY 14853-1801 USA

## Abstract

**Background:**

Plants have two related immune systems to defend themselves against pathogen attack. Initially, pattern-triggered immunity is activated upon recognition of microbe-associated molecular patterns by pattern recognition receptors. Pathogenic bacteria deliver effector proteins into the plant cell that interfere with this immune response and promote disease. However, some plants express resistance proteins that detect the presence of specific effectors leading to a robust defense response referred to as effector-triggered immunity. The interaction of tomato with *Pseudomonas syringae* pv. *tomato* is an established model system for understanding the molecular basis of these plant immune responses.

**Results:**

We apply high-throughput RNA sequencing to this pathosystem to identify genes whose expression changes specifically during pattern-triggered or effector-triggered immunity. We then develop reporter genes for each of these responses that will enable characterization of the host response to the large collection of *P. s.* pv. *tomato* strains that express different combinations of effectors. Virus-induced gene silencing of 30 of the effector-triggered immunity-specific genes identifies *Epk1* which encodes a predicted protein kinase from a family previously unknown to be involved in immunity. Knocked-down expression of *Epk1* compromises effector-triggered immunity triggered by three bacterial effectors but not by effectors from non-bacterial pathogens. Epistasis experiments indicate that Epk1 acts upstream of effector-triggered immunity-associated MAP kinase signaling.

**Conclusions:**

Using RNA-seq technology we identify genes involved in specific immune responses. A functional genomics screen led to the discovery of Epk1, a novel predicted protein kinase required for plant defense activation upon recognition of three different bacterial effectors.

**Electronic supplementary material:**

The online version of this article (doi:10.1186/s13059-014-0492-1) contains supplementary material, which is available to authorized users.

## Background

Plants are vulnerable to attack by many pathogenic microorganisms. To respond to these assaults, plants have evolved two interlinked layers of immunity. Plants initially use pattern recognition receptors to recognize microorganisms by detecting certain conserved features referred to as microbe- or pathogen-associated molecular patterns (MAMPs or PAMPs) [1,2]. Such pattern-triggered immunity (PTI) leads to production of reactive oxygen species, activation of mitogen-activated protein kinase (MAPK) cascades, changes in the intracellular calcium concentration and transcriptional reprogramming [3-5]. However, pathogens such as *Pseudomonas syringae* undermine PTI by delivering virulence proteins (effectors) into the plant cell using a type III secretion system [3]. In a further evolutionary step, some plants acquired intracellular proteins that detect, either directly or indirectly, the presence of specific effectors. This layer of defense, termed effector-triggered immunity (ETI), is often associated with localized programmed cell death (PCD) called the hypersensitive response that may limit pathogen spread [3,6,7].

The interaction between tomato (*Solanum lycopersicum*) and *Pseudomonas syringae* pv. *tomato* (*Pst*), the causative agent of bacterial speck disease, has been extensively used to study the molecular basis of host responses to bacterial infection [8,9]. Among the bacterial MAMPs perceived by tomato, the best characterized derive from the flagellin protein encoded by the *fliC* gene. This protein, which forms the flagellum and therefore plays a key role in motility, possesses two MAMPs that can be detected by tomato: flg22 perceived by the FLS2 receptor [10,11]; and flgII-28, which is recognized by an unidentified receptor referred to as FLS3 [12]. We have recently reported that flagellin-derived MAMPs in *Pst* are the primary elicitors of PTI in tomato, resulting in extensive transcriptional changes [13]. *Pst* strain DC3000 translocates approximately 30 effectors into plant cells and two of these, AvrPto and AvrPtoB, act early in the tomato-*Pst* interaction by interfering with pattern recognition receptor functions and thereby suppressing PTI and promoting bacterial virulence [13-15].

Certain wild relatives of tomato have evolved a specific ETI mechanism to recognize and respond to the presence of AvrPto or AvrPtoB in the plant cell. This mechanism involves members of the Pto kinase family which physically interact with these effectors and act with the nucleotide binding-leucine-rich repeat (NB-LRR) protein Prf to activate ETI [8,16,17]. *Prf* is embedded within the *Pto* family gene cluster on chromosome 5 and this region has been introgressed into many tomato cultivars to confer resistance to bacterial speck disease [8,18]. Changes in gene expression that occur during Pto/Prf-mediated ETI in response to AvrPto were previously analyzed using GeneCalling, an mRNA profiling technology [19]. This study was limited by the lack of a tomato genome sequence and gene annotation, but nevertheless identified 432 ETI-induced genes, including members of 11 transcription factor gene families. The experimental design used in this study did not allow the determination of whether any of these genes were also induced during PTI.

Several recent reports have used microarrays and a series of *Arabidopsis* mutants to analyze changes that occur during ETI and PTI [20,21]. Although PTI involves recognition of MAMPs and ETI recognition of effectors, these studies found that a majority of genes whose expression changes during the defense response are affected by both ETI and PTI [22-25]. Analysis of transcriptome changes also indicated that the PTI response was transient and more vulnerable to being undermined by the pathogen, whereas the ETI response was more prolonged and robust [20]. These differences appeared to be due to how ETI and PTI use the same signaling networks rather than to their use of distinct signaling networks [25]. In the case of PTI, gene expression changes were characterized as being synergistic and for this reason more vulnerable to pathogen suppression. ETI, however, uses the same signaling network in a compensatory way, making it difficult for the pathogen to interfere with this response [25]. Overall, these studies have provided important insights into the dynamics and temporal aspects of plant immunity.

RNA-Seq is a powerful high-throughput technology that is being broadly used for transcriptome studies in different cells and treatments [26-29]. The RNA-Seq approach has recently been applied to the analysis of PTI in the tomato-*Pst* system, where it allowed the identification of a subset of genes whose expression is induced by flagellin-derived MAMPs but reduced by the activity of AvrPto and AvrPtoB effectors, referred to as *FIRE* (flagellin-induced, repressed by effectors) genes [13]. These *FIRE* genes were screened by a virus-induced gene silencing (VIGS) approach which identified a cell wall-associated kinase, SlWAK1, that is required for an effective PTI response [13].

Here we describe the use of the tomato-*Pst* system in an experimental design that allowed the identification of host genes whose expression changes specifically in ETI or PTI. We developed reporter genes for each of these responses which will be useful for analyzing the response of tomato to *Pst* mutants that lack combinations of effectors, other virulence factors, or MAMPs. We then focused on a subset of 30 genes whose expression was induced specifically during ETI and used these in a VIGS screen to determine if they play a demonstrable role in ETI against *Pst*. This approach identified a predicted protein kinase, Epk1, that has not previously been implicated in the plant immune response and which may act in a pathway unique to the immune response triggered by bacterial effectors.

## Results

### Analysis of transcriptome modifications during Pto/Prf-mediated effector-triggered immunity in tomato

In order to study the transcriptome changes in tomato during Pto/Prf-mediated ETI, we infiltrated tomato Rio Grande (RG)-PtoR resistant plants (plants that have a functional Pto/Prf signaling pathway, *Pto/Pto*, *Prf/Prf*) and two different susceptible plants: RG-*prf3* and RG-*prf19* (*Pto/Pto*, *prf/prf*), with *Pseudomonas syringae* pv. *tomato* DC3000 (DC3000) (Figure S1A in Additional file 1). The susceptible lines have a non-functional *Prf* gene due to a 1.1 kb deletion or a G-insertion at position 2,584 (which causes a frameshift), respectively [30]. We collected leaf tissue at 4 and 6 h after inoculation (hai) to assess early changes in host gene expression after translocation of DC3000 effectors AvrPto and AvrPtoB, which occur at about 3 hai. The plants were then maintained in the same conditions to observe signs of disease. As expected, RG-PtoR plants did not develop speck disease whereas the RG*-prf* plants did (Figure S1B in Additional file 1).

For the data analysis, we considered a gene to be ‘expressed’ if it had three or more RPKMs (reads per kilobase per million of mapped reads) in at least one of the treatments analyzed. The cutoff used for the comparisons was *P* <0.05 and ≥2-fold expression change [13]. We compared the transcriptome changes observed in resistant (RG-PtoR) and susceptible (RG-*prf3* or RG-*prf19*) plants and found that the number of differentially expressed genes increased from 4 to 6 h (Figure S1C in Additional file 1). We considered a gene to be induced during ETI when its expression was higher in RG-PtoR than in RG-*prf3* or RG-*prf19* plants. On the other hand, a gene was considered to have reduced expression during ETI when it was expressed higher in RG-*prf3* or RG-*prf19* than in RG-PtoR plants after DC3000 inoculation. The number of genes with reduced expression was higher than those with induced expression in both PtoR/*prf3* and PtoR/*prf19* combinations. As expected, the overall gene expression differences of the two susceptible lines were very small, with only 27 differentially expressed genes between RG-*prf3* and RG-*prf19.*

Taking just the ETI-induced genes from this experiment at 6 h, we compared them with PTI-induced genes reported previously [13]. For this purpose we considered as PTI-induced genes those increased by flgII-28, DC3000 Δ*hrcQ-U*, *Pseudomonas fluorescens* or the *Pseudomonas putida* treatments [13]. This comparison revealed that essentially the same number of genes was induced only in ETI, only in PTI, or in both responses (Figure S1D in Additional file 1). Thus, although there was overlap in the ETI- and PTI-associated transcriptomes, there also appeared to be unique gene expression changes associated with each of these immune responses.

### Transcriptome changes associated with Pto/Prf-mediated ETI and flagellin-activated PTI revealed a set of genes specifically induced in each immune response

**Figure 1:**
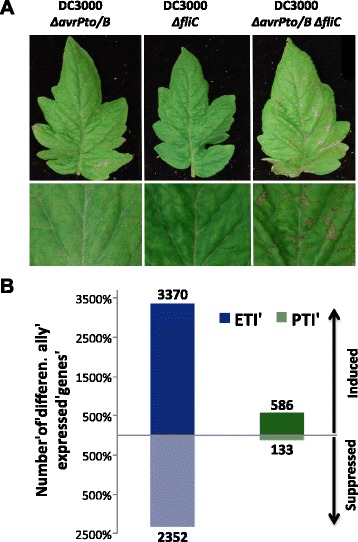
**Transcriptome changes associated with Pto/Prf-mediated ETI and flagellin-activated PTI. (A)** RG-PtoR tomato plants were vacuum-infiltrated with different DC3000 mutants (Table 1). Leaf photographs were taken 4 days later. **(B)** Total number of genes differentially expressed during ETI (calculated as the ratio between the expression in DC3000 Δ*fliC* and DC3000 Δ*avrPto* Δ*avrPtoB* Δ*fliC*) or PTI (calculated as the ratio between DC3000 Δ*avrPto* Δ*avrPtoB* and the triple mutant) at 6 hai. A ≥2-fold difference and *P* <0.05 were used as cutoff. The number of genes in each category is shown.

The above comparison included data from independent experiments that used different bacterial strains and titers. We therefore designed an experiment to assess ETI and PTI in RG-PtoR using a series of *Pst* DC3000 strains which have different mutations that allow dissection of the plant immune response (Table 1). We collected samples at 6 hai and monitored the development of disease in these plants. Plants infiltrated with DC3000 Δ*fliC* had no disease symptoms due to the recognition of AvrPto and AvrPtoB effectors by Pto [8] (Figure 1A). In contrast, when these two effectors were absent, the plants developed speck disease (DC3000 Δ*avrPto* Δ*avrPtoB* and DC3000 Δ*avrPto* Δ*avrPtoB* Δ*fliC* strains). Plants infiltrated with the triple mutant showed the greatest disease severity due to the absence of ETI and flagellin-activated PTI induction (Figure 1A). This experimental design allowed us to identify gene expression changes associated with flagellin-activated PTI (DC3000 Δ*avrPto* Δ*avrPtoB* versus DC3000 Δ*avrPto* Δ*avrPtoB* Δ*fliC*) and Pto/Prf-mediated ETI (DC3000 Δ*fliC* versus DC3000 Δ*avrPto* Δ*avrPtoB* Δ*fliC*).

**Table 1:** **Summary of the experiments performed for the RNA-Seq analysis**

**Plant**	**Strain**	**Concentration**	**Time point**
Tomato	DC3000 Δ*fliC* ^a^	5 × 10^6^ cfu/ml	6 h
RG-PtoR^b^	DC3000 Δ*avrPto* Δ*avrPtoB* ^c^		
	DC3000 Δ*avrPto* Δ*avrPtoB* Δ*fliC* ^d^		

^a^ *Pseudomonas syringae* pv. *tomato* (*Pst*) DC3000 mutant, lacking flagellin. ^b^Tomato Rio Grande-PtoR plants (*Pto/Pto*, *Prf/Prf*). ^c^ *Pst* DC3000 mutant, lacking AvrPto and AvrPtoB effectors. ^d^ *Pst* DC3000 mutant, lacking AvrPto, AvrPtoB and flagellin.

**Figure 2:**
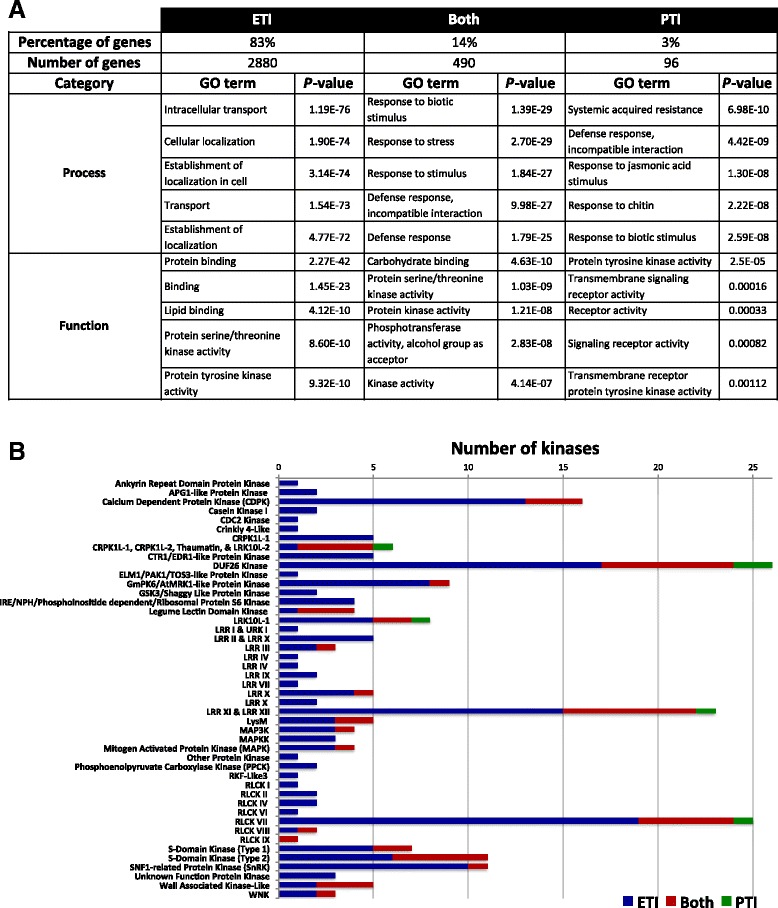
**Transcriptome comparisons of plants treated with different DC3000 mutants revealed the presence of a set of genes that are specifically induced during ETI and PTI. (A)** Summary of genes induced by ETI, PTI, or both. A ≥2-fold difference and *P* <0.05 were used as cutoff. Genes in each category were used for Gene Ontology (GO) term analysis. The top five most-enriched GO terms in the categories process and function are shown. **(B)** The number of induced genes in protein kinase families during ETI, PTI, or both. A ≥2-fold difference and *P* <0.05 were used as cutoffs.

We observed a larger number of ETI- than PTI-associated gene expression changes and, in both cases, more genes were induced (higher expression in the plants infiltrated with DC3000 Δ*fliC* or DC3000 Δ*avrPto* Δ*avrPtoB* than with the triple mutant) than suppressed (higher expression in the triple mutant compared with the other two strains used) (Figure 1B). No differences in bacterial populations were observed at the sampling time (6 hai), indicating that the changes in gene expression are not due to differences in virulence between the DC3000 strains at this time point (Additional file 2). For this study we focused on genes with induced expression and classified them into three categories: ETI-specific, PTI-specific or induced during both immune responses. From the total number of induced genes, 83% were ETI-specific, 14% were shared between both immune responses and 3% were PTI-specific (Figure 2A). Details of the genes in each category are provided in Additional file 3. We examined the induced genes for their transcript abundance (maximum and average RPKM) and fold change (Additional file 4). Although we did not observe differences in gene induction levels between ETI and PTI (fold-change; Figure S3C in Additional file 4), we found that the majority of PTI-specific genes had a maximum of 10 RPKM, whereas the majority of ETI-specific genes had a maximum of 50 RPKM (Figure S3A in Additional file 4), indicating that transcript levels of ETI-specific genes are generally higher than those of PTI-specific ones.

We performed a bibliographic search to identify genes in the three categories that have been described previously as being implicated in plant-pathogen interactions (Additional file 3). Approximately 25% of the genes had at least one publication associating them with plant immunity (Additional file 3). In the ETI-specific category, we found 10 genes that had been previously confirmed to be involved in ETI mediated by the Pto kinase using a loss of function approach [9].

### Gene Ontology term analysis supports the central importance of protein kinase activity in plant immunity

We performed a Gene Ontology (GO) term analysis using the tomato genome sequence as a reference (Figure 2A; Additional file 5). The ‘Process’ GO terms of ‘defense’ or ‘response to stimulus’ or ‘stress’ were the most common for the PTI-specific and the shared PTI/ETI genes, whereas ‘transport’ and ‘cellular localization’ had the highest enrichment for the ETI-specific genes. Additional Process GO terms associated with ETI were ‘response to stress’, ‘response to stimulus’, ‘signal transduction’ and ‘regulation of cell death’ (Additional file 5). In the ‘Function’ category of GO terms all three groups of genes showed similar results with ‘protein kinase activity’ and ‘lipid-binding’, ‘carbohydrate-binding’, or ‘protein-binding‘ being most prominent. Among the ETI-specific genes, 176 of them are kinases representing 27% of the total expressed kinases in our analysis (657 expressed kinases out of 1,150 total predicted in tomato [31]), supporting the relevance of protein kinases in plant immunity.

We categorized the protein kinase-encoding genes whose expression was induced by our treatments (Figure 2B). Of the 64 protein kinase families present in tomato [31], 46 (72%) had a member that was induced in at least one of the categories (ETI-specific, both, or PTI-specific). PTI-specific kinases occurred in just five families and all of them also contained ETI-specific kinases. A total of 20 kinase families have genes induced during both PTI and ETI. The remaining 26 are families that possess only ETI-specific induced kinases and many of these have low numbers of induced kinase genes (from 1 to 5).

We also categorized induced transcription factor genes and found that a total of 249 occurred in all three groups, with 190 being induced specifically during ETI (Figure S4A in Additional file 6). Analysis of the transcription factor families indicated that those with the largest number of induced genes (*AP2-EREBP*, *C2H2*, *MYB*, *NAC* and *WRKY*) contain genes from all the three categories (ETI-specific, common genes and PTI-specific). However, most of the families have only ETI-specific induced transcription factors (Figure S4B in Additional file 6).

### Development of Pto/Prf-mediated ETI and flagellin-activated PTI reporter genes

**Figure 3:**
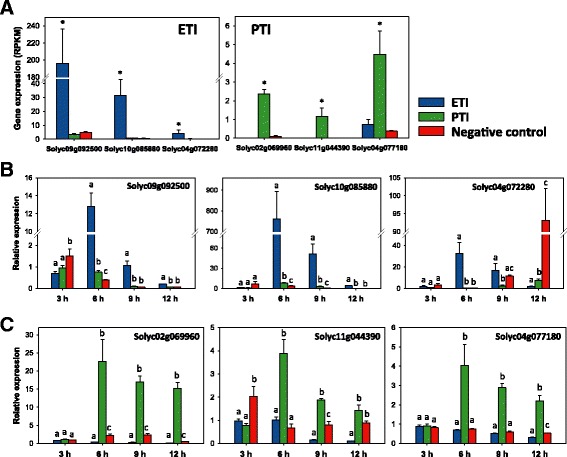
**Development of Pto/Prf-mediated ETI and flagellin-activated PTI reporter genes. (A)** Transcript abundance (RPKM) of selected reporter genes in RG-PtoR at 6 hai with DC3000 strains as in Figure 1. Asterisks indicate significant differences (*P* <0.05) with raw *P*-values corrected for multiple testing using the false discovery rate. **(B,C)** Relative expression (based on quantitative RT-PCR) of ETI- **(B)** and PTI-reporter genes **(C)** at different time points after inoculation with DC3000 strains as in Figure 1 to induce ETI or PTI. *SlATPase* was used as the reference gene for quantitative RT-PCR; similar results were obtained using *SlCBL1* (Calcineurin B-like protein) and *SlEF1*α. Bars represent the mean of four biological replicates with their corresponding standard error. Different letters indicate significant differences at the same time point using Tukey’s HSD test (*P* <0.01). RPKM, reads per kilobase per million of mapped reads.

We identified and tested genes that are specifically induced during either ETI or PTI with the objective of developing reporters that will allow distinguishing between cell death and other responses associated with disease or ETI, which can be phenotypically similar. To achieve this, we selected three ETI- and three PTI-specific genes from our RNA-Seq data. Genes encoding a UDP-glucosyltransferase (Solyc09g092500, Solyc10g085880) or a laccase (Solyc04g072280) were chosen because they showed high induction only during ETI (Figure 3A). Genes encoding a NAC domain protein (Solyc02g069960), an osmotin-like protein (Solyc11g044390) and a potential lipid particle serine esterase (Solyc04g077180) were selected as PTI-specific markers (Figure 3A).

RG-PtoR plants were again infiltrated with different DC3000 strains to assess the response of the reporter genes to ETI and PTI, but in this case we took leaf samples at 3, 6, 9 and 12 hai to determine the temporal pattern of their induction. By using qRT-PCR we confirmed each gene to be ETI- or PTI-specific at 6 hai as found from the RNA-Seq data (Figure 3B,C). In the case of the ETI-induced genes, the two UDP-glucosyltransferase genes were shown to be good reporters at 6, 9, and 12 hai. The laccase gene was ETI-specific only at 6 h after treatment (Figure 3B). All three PTI-specific genes were induced at each time point only during this immune response starting at 6 hai (Figure 3C). None of the six genes proved to be effective at distinguishing the two immune responses at 3 hai, which is likely before translocation of the effectors into the plant cell.

### Identification of a novel kinase involved in plant immunity using a virus-induced gene silencing screen

To determine if the ETI-specific genes play a demonstrable role in this immune response we selected a subset of 30 genes that encode predicted protein kinases and transcription factors and performed a loss-of-function screen using VIGS in *Nicotiana benthamiana* (Additional file 3). Leaves of silenced plants were infiltrated with a mix of *Agrobacterium tumefaciens* strains carrying 35S:*Pto* or 35S:*avrPto*. Three genes with known roles in ETI were included as positive controls (*SlMAPKKK*α, *SlMEK2* and *SlPrf*; Additional file 7) [18,32,33]. Negative controls were a fragment of the green fluorescent protein (*GFP*) gene and an *Escherichia coli*-derived DNA fragment (*Ec1*) (Additional file 7).

**Figure 4:**
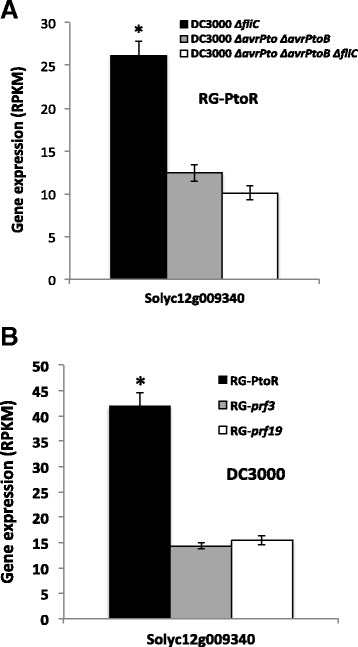
**Transcript abundance (RPKM) of** ***SlEpk1*** **. (A)** RNA-Seq expression analysis of *SlEpk1* gene in tomato RG-PtoR plants infiltrated with different DC3000 strains. **(B)** Resistant (RG-PtoR) and susceptible (RG-*prf3* and RG-*prf19*) tomato plants infiltrated with DC3000. Leaf samples were taken at 6 h after induction. Bars represent the mean of three biological replicates with their corresponding standard deviation. Asterisks indicate significant differences (*P* <0.05) with raw *P*-values corrected for multiple testing using the false discovery rate.

From this screen, we identified a predicted protein kinase-encoding gene whose silencing delayed PCD elicited by Pto/AvrPto (Figure S5A in Additional file 8). This protein is annotated as a serine/threonine tyrosine protein kinase (Solyc12g009340) that belongs to the GmPK6/AtMRK1-like protein kinase family [31]. SlEpk1 is a small protein (401 amino acids) that has a predicted protein kinase domain between residues 120 and 382. No other motifs or localization signals were found outside the kinase domain. The gene is induced specifically during ETI in both of the RNA-Seq experiments that we performed (Figure 4A,B) and plants silenced with the same construct were not reduced in flg22-induced reactive oxygen species (ROS) production (Figure S5B in Additional file 8). Therefore, we refer to it as *SlEpk1*, for *Solanum lycopersicum* ETI-specific protein kinase 1. The GmPK6/AtMRK1-like protein kinase family consists of 20 members in *Arabidopsis*, 30 in tomato and 42 in *N. benthamiana* [31]. A phylogenetic analysis using proteins from these three species revealed that SlEpk1 has two orthologs in tetraploid *N. benthamiana* (NbS00051202g0009.1 and NbS00020954g0005.1) (Additional file 9).

### Silencing effectiveness of the *SlEpk1* virus-induced gene silencing construct in *N. benthamiana*

A VIGS construct using a 260 bp fragment of the tomato *Epk1* gene was used for silencing *N. benthamiana* plants (Additional file 7). To analyze the specificity of the silencing, we first identified genes in *N. benthamiana* that have 100% nucleotide identity to the *Epk1* fragment over a stretch of ≥17 nucleotides. Twelve such genes were identified, although six were not considered further due to low or no expression (based on *N. benthamiana* RNA-Seq data) or because they appeared to derive from a sequence encoding a non-functional protein (Additional file 10). We performed qRT-PCR using *Ec1*- and *SlEpk1*-silenced *N. benthamiana* plants on the six remaining genes (Additional file 10). Of these six genes, transcript abundance for four was reduced in the *SlEpk1*-silenced plants, with the degree ranging from 20 to 80% (Figure S5C in Additional file 8). It was notable that the more similar the *N. benthamiana* gene sequence is to *SlEpk1*, the better it was silenced (Figure S5C in Additional file 8; Additional file 11). All four of the silenced genes are in the same clade with *SlEpk1* (Additional file 11, clade A); the two unaffected genes belong to another clade (Additional file 11, clade B). These results indicate that one or more genes in *N. benthamiana* are *Epk1*-related genes and one or more of them could contribute to ETI.

### Silencing of *SlEpk1* compromises resistance to *Pseudomonas syringae* pv. *tabaci* (AvrPto)

**Figure 5:**
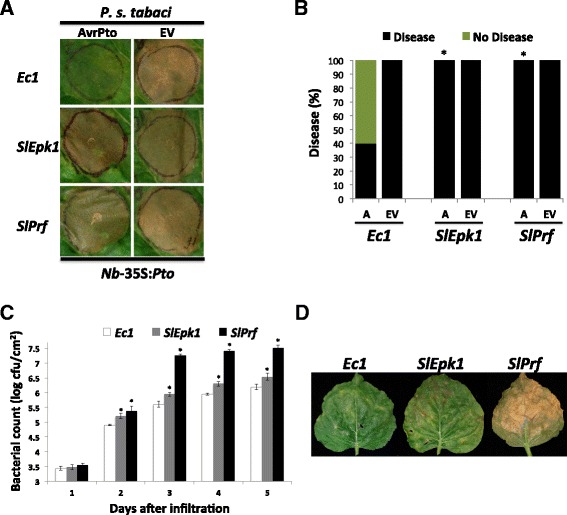
**Silencing of** ***SlEpk1*** **compromises resistance to** ***Pseudomonas syringae*** **pv.** ***tabaci*** **(AvrPto).** **(A)** *N. benthamiana* (*Nb*) 35S:*Pto* plants were silenced for the genes shown and subsequently syringe-infiltrated with 5 × 10^4^ cfu/ml *P. s.* pv. *tabaci* expressing *avrPto* (A) or empty vector (EV). Photographs were taken 4 days after inoculation. **(B)** Percentage of the infiltrated leaf circles that developed disease. Asterisks indicate significant differences compared with *Ec1*-silenced plants using Fisher’s exact test (*P* <0.05). Six plants were used per silencing construct. **(C)** *P. s. tabaci* (*avrPto*) populations in leaves. Leaves of silenced plants were infiltrated with 6 × 10^4^ cfu/ml *P. s. tabaci* (*avrPto*) and sampled to measure bacterial populations at the times shown. Bars represent the mean of six plants per construct with their corresponding standard error. Asterisks indicate significant differences compared with *Ec1*-silenced plants using a Student’s *t*-test (*P* <0.05). **(D)** Disease lesions at 9 days after infiltration; *Ec1*, *Escherichia coli* fragment 1 (negative control).

To further investigate the role of *SlEpk1* in Pto/Prf-mediated ETI, we silenced the gene in *N. benthamiana* plants that express *Pto* (Nb-35S:*Pto*) and included control plants silenced for *Ec1* or *SlPrf*. Leaves of the silenced plants were infiltrated with a low titer (5 × 10^4^ cfu/ml) of *P. s. tabaci* strains expressing AvrPto or an empty vector [34]. As expected, all of the plants infiltrated with *P. s. tabaci* carrying the empty vector developed disease (Figure 5A,B). The *Ec1* plants infiltrated with *P. s. tabaci* carrying AvrPto did not develop disease due to the recognition of AvrPto by Pto and activation of ETI. Plants silenced for *SlEpk1*, or the positive control *SlPrf*, developed disease upon infiltration with *P. s. tabaci* expressing AvrPto (Figure 5A,B). As a further test, we vacuum-infiltrated silenced plants with *P. s. tabaci* AvrPto to measure bacterial populations. Bacteria reached higher numbers in plants silenced for *SlEpk1* or *SlPrf* compared with the *Ec1* control (Figure 5C). Additionally, plants silenced for *SlEpk1* or *SlPrf* developed more severe disease symptoms (Figure 5D). These results indicate that *SlEpk1* participates in the Pto/Prf pathway in *N. benthamiana.*

### *SlEpk1* also plays a role in effector-triggered immunity activated by the bacterial effector HopQ1-1

**Figure 6:**
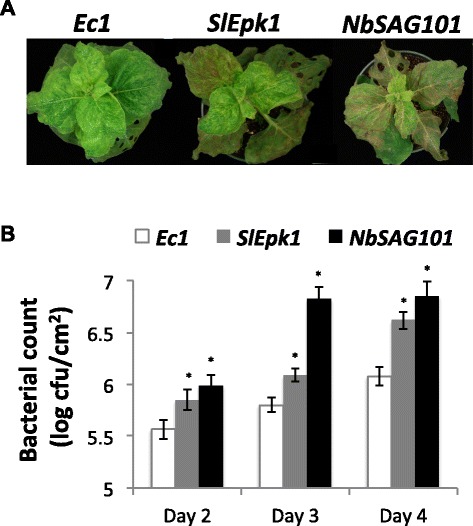
***SlEpk1-*** **silenced plants are compromised in resistance elicited by HopQ1-1 recognition in** ***N. benthamiana*** **. (A)** *N. benthamiana* plants, silenced for the genes shown, were vacuum-infiltrated with 5 × 10^4^ cfu/ml *P. s.* pv. *tabaci* expressing the effector HopQ1-1. Photographs of plants were taken 5 days after infiltration. **(B)** *P. s. tabaci* (*hopQ1-1*) populations in leaves. Silenced plants were infiltrated with 5 × 10^4^ cfu/ml *P. s. tabaci* (*hopQ1-1*) and sampled to measure bacterial populations. Bars represent the mean of six plants per construct with their corresponding standard error. Asterisks indicate significant differences compared to *Ec1*-silenced plants using a Student’s *t*-test (*P* <0.05). *Ec1*, *Escherichia coli* fragment 1 (negative control); *NbSAG101* (positive control that compromises ETI).

To investigate whether or not SlEpk1 plays a role exclusively in ETI activated by AvrPto recognition, we silenced the *Ec1*, *SlEpk1* or *NbSAG101* gene in *N. benthamiana* plants (Additional file 7) and vacuum-infiltrated them with *P. s. tabaci* expressing HopQ1-1, a strain that activates ETI in *N. benthamiana* [35]. *NbSAG101* was found in previous VIGS experiments to delay the development of PCD caused by DC3000 or *P. s. tabaci* HopQ1-1 (H Rosli and M Pombo, unpublished). The VIGS construct was designed based on two orthologs of the tomato *SAG101* in *N. benthamiana* (NbS00037653g0001.1 and NbS00039736g0001.1). *SlEpk1*- and *NbSAG101*-silenced plants developed more disease symptoms than *Ec1* plants, supporting a role for *Epk1* in ETI activated by HopQ1-1 (Figure 6A). Consistent with the enhanced disease, *P. s. tabaci* (HopQ1-1) reached higher population levels in plants silenced with *SlEpk1* and *NbSAG101* (Figure 6B) compared with the *Ec1* control, confirming that these plants are compromised in the ETI response triggered by HopQ1-1 recognition.

### *SlEpk1* does not contribute to ETI activated by several non-bacterial effectors

**Figure 7:**
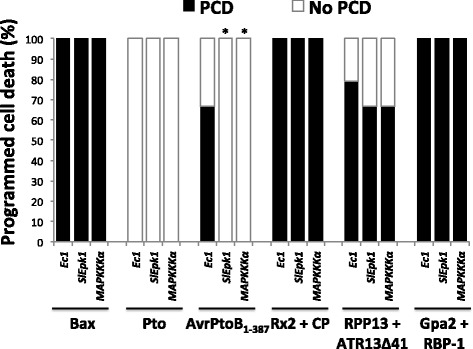
***SlEpk1*** **contributes to programmed cell death associated with bacterial effector-plant R protein interaction.** *N. benthamiana* silenced plants were syringe-infiltrated with *A. tumefaciens* carrying different effector/R gene pairs to elicit PCD. PCD percentage was calculated as described in the Materials and methods using 12 plants per construct. Asterisks indicate significant differences compared with *Ec1*-silenced plants using Fisher’s exact test (*P* <0.01). *Ec1*, *Escherichia coli* fragment 1; Pto, resistance to *Pseudomonas syringae* pv. *tomato*; Bax, Bcl-2-associated X protein; AvrPtoB_1–387_, AvrPtoB lacking the E3-ligase domain; Rx2, resistance to potato virus X (PVX); CP, coat protein of PVX; RPP13, recognition of *Hyaloperonospora parasitica* 13; ATR13Δ41, *Arabidopsis thaliana-*recognized 13 with 41 amino acids of the signal peptide deleted; Gpa2, *Globodera pallida* 2; RBP-1, Ran-binding protein 1.

To further characterize SlEpk1 involvement in PCD associated with ETI, we tested effector/R gene pairs derived from diverse plant-pathogen interactions. We silenced *Ec1*, *SlEpk1* and *MAPKKK*α in *N. benthamiana* and syringe-infiltrated leaves with different ETI activators: AvrPtoB_1–387_, a truncated bacterial effector [36]; potato (*Solanum tuberosum*) Rx2/coat protein of PVX [37]; *Arabidopsis* RPP13/ATR13^Emco5_^Δ41aa from the oomycete *Hyalosperonospora arabidopsidis* [38]; and potato Gpa2/RBP-1 from the potato cyst nematode [39]. Bax, a murine Bcl-2-associated X protein, was also included as it triggers cell death in plants [40], and Pto without an effector was included as a negative control. With the exception of AvrPtoB_1–387_, PCD associated with each of these elicitors was unaffected in *SlEpk1*-silenced plants (Figure 7), suggesting that *SlEpk1* may play a role only in bacterial immunity-associated PCD in *N. benthamiana*.

### SlEpk1 appears to act upstream of effector-triggered immunity-associated MAPK signaling

To investigate the position at which SlEpk1 might act in the signaling pathway activated upon AvrPto recognition by Pto, we performed an epistasis experiment that involved silencing and transiently expressing genes known to be involved in ETI [32]. *N. benthamiana* plants were silenced for *SlEpk1*, *MAPKKK*α, *MEK2* or *SlPrf*, with *Ec1* as a negative control. MAPKKKα and MEK2^DD^, both of which cause ETI-associated PCD, were then transiently expressed under an estradiol inducible promoter in the silenced leaves. If a silenced gene acts downstream or directly with MAPKKKα or MEK2, we expect to see compromised PCD in these experiments.

**Figure 8:**
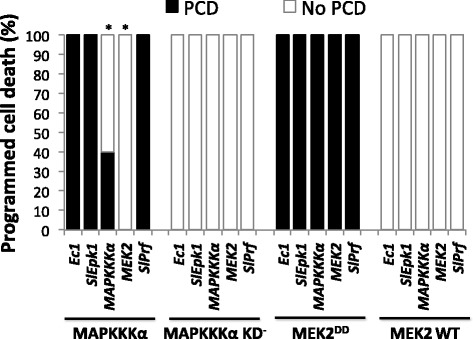
**SlEpk1 appears to act upstream of MAPK signaling.** Leaves of *N. benthamiana* plants silenced for *Ec1*, *SlEpk1*, *MAPKKK*α, *MEK2* or *SlPrf* were syringe-infiltrated with *Agrobacterium* carrying transgenes for MAPKKKα, MAPKKKα KD^-^, MEK2^DD^ or MEK2 WT. PCD percentage was calculated as described in the Materials and methods using six plants per construct. Asterisks indicate significant differences compared with *Ec1*-silenced plants using Fisher’s exact test (*P* <0.05). PCD, programmed cell death; MEK2^DD^, constitutive-active MAP kinase kinase 2; MEK2 WT, wild type MEK2; MAPKKKα KD^-^, MAPKKKα with an inactive kinase domain.

As expected, expression of MAPKKKα or MEK2 in *Ec1*-silenced plants resulted in full PCD (Figure 8). The same result was obtained for the silenced genes using MEK2^DD^, indicating that none of these genes act downstream or directly with MEK2 in the signaling cascade (Figure 8). PCD induced by MAPKKKα was delayed in *MAPKKK*α*-*silenced plants as expected and was abolished by silencing of the downstream factor *MEK2*. Silencing of *SlEpk1* or *SlPrf* had no effect on the PCD induced by MAPKKKα or MEK2^DD^, indicating that SlEpk1 acts upstream of this MAPK cascade or that it functions independently of this MAPK pathway (Figure 8).

## Discussion

We discovered in two independent experiments that there are plant genes whose expression is induced specifically during ETI or PTI. There are previous reports describing an overlap in transcriptomic changes during ETI/PTI [22,23] and we also observed that a majority of the genes whose expression is altered at 6 h after activation of PTI are also altered at the same time point after activation of ETI. However, we identified a high percentage of ETI-induced genes that were not affected during PTI. The significant differences we observed between ETI and PTI could be due to more robust signaling in the case of ETI and because the delivery of effectors into the plant cell suppresses flagellin-activated PTI [41]. Previous studies have not focused on immune response-specific gene expression changes as we have done here, but have instead looked for shared signaling networks between ETI and PTI [22-25]. The discovery of ETI- and PTI-specific genes raises the possibility that these distinct classes are enriched for genes that play a role in these specific immune responses and our bibliographic search supported this hypothesis. In agreement with this, our screening of just 30 ETI-specific genes successfully identified a predicted protein kinase from a family previously unknown to have a role in the plant immune response.

Our GO term analysis revealed characteristics about the ETI- and PTI-specific gene sets that distinguish them from each other. For example, the PTI-specific group is enriched in secondary metabolite biosynthetic processes mainly related to the phenylpropanoid pathway known to be important in plant defense. Although it is not exclusive to PTI, membrane receptor kinase activity seems to have a primary role in this form of plant immunity. As expected, only in the ETI-specific gene group is there an enrichment in processes related to the hypersensitive response [42,43], such as programmed cell death, ion homeostasis, regulation of apoptotic process, response to hydrogen peroxide and other ROS, membrane organization and vesicle-mediated transport. A number of genes described previously as involved in incompatible plant-pathogen interactions were found to be specifically induced during ETI, supporting the idea that gene expression differences are a useful way to distinguish the two immune responses [9].

Several previous studies have used reporter genes as a readout for activation of PTI and its subsequent suppression by pathogen effectors [44-47]. Reporter genes for PTI have also been developed for tomato and *N. benthamiana* [5,48,49]. However, these reporters have typically not been shown to be specific for PTI and may also be induced during ETI. In fact, using our RNA-Seq data we found that three tomato PTI reporter genes developed previously [5] are also induced during ETI. In addition, the tomato genes most closely related to *FRK1*, a commonly used PTI reporter gene in *Arabidopsis*, are not expressed or not induced during ETI or PTI, highlighting the importance of plant genera- or species-specific reporter genes. Plant-pathogen interactions can result in host cell death due to ETI or disease that is associated with activation and subsequent suppression of PTI. Reporter genes that distinguish between these two immune responses will be a valuable tool to characterize the plant response to *Pst* strains that have mutations affecting specific effectors, other virulence factors or MAMPs. Our RNA-Seq data and subsequent qRT-PCR analyses identified six such reporter genes for tomato that can be used as specific plant defense markers in a biologically relevant time frame after bacterial inoculation (from 6 to 12 hai). In the future, development of specific reporter genes for *N. benthamiana* will be useful for understanding the contribution of SlEpk1 to ETI.

Our loss-of-function approach to screening a set of 30 ETI-specific genes identified a novel predicted protein kinase, SlEpk1. This protein is most closely related to the GmPK6/AtMRK1-like protein kinase family. In *Arabidopsis*, this family of kinases is in the MAPKKK group of proteins [50]. However, in tomato it is considered as an independent family with around 51 members [31]. In both *Arabidopsis* and tomato, the genes in this family are known only from genomic analysis without any reports about their role in a specific biological process.

There are many examples of proteins that play a role in ETI against diverse pathogens. For example, RAR1 has been shown to act in an ETI pathway triggered by many coiled-coil NB-LRR proteins and EDS1 acts in pathways triggered by many different TIR-NB-LRR R proteins [51,52]. Similarly, silencing of the genes encoding MAPKKKα, MEK2 or TFT7, a 14-3-3 protein, compromises ETI elicited by R proteins directed at diverse pathogens [32,33,53,54]. In contrast, our experiments with five different R protein/effector pairs indicate that SlEpk1 may act in a pathway that responds specifically to *Pst* effectors. If future experiments confirm this specificity, it suggests that Epk1 may act early in these ETI pathways. Consistent with the apparent specificity of SlEpk1 for bacterial ETI, we discovered that this kinase likely acts upstream of MAPKKKα. It is possible that Epk1 participates in a complex directly with Pto/Prf and in a protein complex involved in HopQ1-1 recognition. Alternatively, SlEpk1 might act downstream of these recognition complexes at a juncture shared by bacterial resistance pathways, possibly even interacting with MAPKKKα.

Further experiments are needed to understand the function of SlEpk1 in plant immunity. For example, it will be important to determine if SlEpk1 is an active kinase and if its kinase activity is required for its function in plant defense triggered by bacterial effectors. If SlEpk1 is an active kinase, it will be important to determine its possible substrates. SlEpk1 and its closest tobacco and *Arabidopsis* genes are described as serine/threonine tyrosine protein kinases [55,56]. In the case of tobacco, DSK1 was confirmed to be phosphorylated on all three of these amino acids [55]. Lately, tyrosine phosphorylation of EF-TU RECEPTOR (EFR) upon MAMP recognition was shown to play an important role in innate immunity [57]. Consequently, if Epk1 is an active kinase, it will be interesting to determine which amino acids in its kinase domain are phosphorylated. An Epk1-like protein has been reported to localize to the chloroplast in tobacco and to the plasma membrane in *Arabidopsis* [55,58]. Determining the subcellular localization of the tomato SlEpk1 protein may also provide insights into the role of SlEpk1 in plant immunity.

## Conclusions

Using RNA-Seq technology, we identified genes whose transcript abundance is increased specifically during PTI or ETI. Highlighting the relevance of these sets of induced genes, 25% of them were implicated previously as contributing to plant-pathogen interactions. We used these data to develop specific reporter genes that will be useful for future studies of plant responses to different bacterial pathogens. A functional genomics screen identified a predicted protein kinase, Epk1, as playing a role in ETI response to three different bacterial effectors. The ETI- and PTI-specific gene sets provide a unique resource for further dissection of the plant immune responses.

## Materials and methods

### Bacterial strains and growth conditions

*Pseudomonas* strains were grown on King’s B medium at 30°C. *A. tumefaciens* and *E. coli* were grown on Luria-Bertani (LB) medium at 30°C and 37°C, respectively. Antibiotics used were: ampicillin (100 μg/ml), kanamycin (50 μg/ml), rifampicin (10 μg/ml), spectinomycin (50 μg/ml) and gentamycin (10 μg/ml). Bacterial strains are listed in Additional file 12.

### Plant material and bacterial infiltrations

To analyze changes in transcript abundance during ETI, 4-week old resistant Rio Grande-PtoR (RG-PtoR), susceptible Rio Grande-*prf3* (RG-*prf3*) or Rio Grande-*prf19* (RG-*prf19*) plants were vacuum-infiltrated with a suspension of 2 × 10^7^ cfu/ml of *Pseudomonas syringae* pv. *tomato* (*Pst*) DC3000. The experiment was repeated in three successive weeks (three biological replicates) and leaf samples were collected in each experiment at 4 and 6 hai, frozen in liquid N_2_ and stored at -80°C until processed.

For comparisons between ETI and PTI, RG-PtoR plants were vacuum-infiltrated with 5 × 10^6^ cfu/ml of different DC3000 mutants (DC3000 Δ*hopQ1-1* Δ*fliC*, DC3000 Δ*hopQ1-1* Δ*avrPto* Δ*avrPtoB* and DC3000 Δ*hopQ1-1* Δ*avrPto* Δ*avrPtoB* Δ*fliC*) (Table 1). Leaf samples from infiltrations performed in three successive weeks (three biological replicates) were taken at 6 hai and manipulated as described above. For all experiments, after the samples were collected plants were maintained in the same conditions to observe disease symptoms (3 to 4 days after infiltration).

### RNA-Seq library preparation and analysis

Total RNA was isolated with the Plant RNA isolation reagent (Life Technologies, Grand Island, NY, USA) according to the manufacturer’s instructions. Libraries for sequencing were constructed as described in [59] except that barcode sequences were in the reverse PCR primer. Barcoded libraries were multiplexed by 12 or 15 in each lane and sequenced on an Illumina HiSeq 2000 system using the single-end mode. The length of the reads was around 51 bp. Detailed information about the quality of reads in each replicate is provided in Additional file 13. Analysis of the RNA-Seq data was performed as described in [13]. Genes were considered to be induced when the expression was higher in RG-PtoR than in RG-*prf3* or RG-*prf19* plants infiltrated with DC3000; or when the expression was higher in RG-PtoR plants inoculated with DC3000 Δ*fliC* or DC3000 Δ*avrPto* Δ*avrPtoB* compared with DC3000 Δ*avrPto* Δ*avrPtoB* Δ*fliC*. In the opposite situation the genes were considered to be suppressed. Sequence reads have been deposited in the NCBI Sequence Read Archive (SRA) under accession number SRP043126 and SRP043127. Processed data are available from the Tomato Functional Genomics Database [60] (accessions D010 and D011).

### Development of effector-triggered and pattern-triggered immunity reporter genes

RG-PtoR plants were vacuum-infiltrated with the DC3000 strains as shown in Table 1. Leaf tissue was collected from four biological replicates at 3, 6, 9 and 12 hai. Total RNA was isolated using the Plant RNA isolation reagent (Life Technologies) following the manufacturer’s instructions. Total RNA (8 μg) was processed with TURBO DNA-free kit (Life Technologies) for 60 minutes at 37°C. After DNase treatment, 4 μg RNA was used to prepare cDNA using SuperScript III First-Strand Synthesis System (Life Technologies) with oligo(dT)_20_. qRT-PCR was performed as described previously [5]. The sequences of the primers used were: Solyc09g092500 F: 5′-TTGGACAGATCAAGGGACTAATG -3′, R: 5′-CACTCTCAACCACACCATCTT-3′; Solyc10g085880 F: 5′-CCTGGATTGTTCGACAAGAT-3′, R: 5′-CTCCTCCGCTTTCTTCATTT-3′; Solyc04g072280 F: 5′-AACGTCCCGATCGTAGAA-3′, R: 5′-GGATGATCAACTCCACCTAATAA-3′; Solyc02g069960 F: 5′-AGCCAACAAAGCTCAGGAA-3′, R: 5′-CATCCCAGTTGCCATGTTCTA-3′; Solyc11g044390 F: 5′- TCCTAATGACTTGTCCGGATTT-3′, R: 5′-AGTATCACTAGGGCAAGCAAATA-3′; Solyc04g077180 F: 5′-CAGCATTCTGTGGGCTATAC-3′, R: 5′- CCGAAGAAGAAGAGGTTTCC-3′. Data were normalized using: *SlATPase* (Solyc04g081090) F: 5′-TTGCTGAAGCCTTGGCTCTTTACG-3′, R: 5′-ACCAGCGCGAGAAGAAAGGATGAT-3′; *SlEF1*α (Solyc06g005060) F: 5′-TCCAAAGATGGTCAGACCCGTGAA-3′, R: 5′-ATACCTAGCCTTGGAGTACTTGGG-3′ and *SlCBL1* (Solyc12g015870) F: 5′-CCATCCAAATGCTCCGATCGATGA-3′, R: 5′-TGCCTCTCAATGAAGCCTTGTTGC-3′. Cycling conditions during qRT-PCR were 50°C for 2 minutes, 95°C for 10 minutes, and 40 cycles of 95°C for 30 s, 55°C for 30 s and 72°C for 30 s. A Tukey’s HSD test (*P* <0.01) was used for statistical analysis of the results.

### Virus-induced gene silencing

Tomato genes for which a clear ortholog could be identified in *N. benthamiana* were selected for designing VIGS constructs. Fragments of 280 to 350 bp were chosen using a VIGS tool [61]. Primers were designed inside this region using Primer3 [62]. PCR amplification was performed using cDNA obtained from RG-PtoR leaves tissue infiltrated with 2 × 10^7^ cfu/ml DC3000 or *N. benthamiana* 35S:Pto plants infiltrated with 2 × 10^7^ cfu/ml DC3000 Δ*hopQ1-1*. PCR products were cloned into pCR8/GW/TOPO vector (Life Technologies) and recombined into a Gateway compatible TRV2 vector [63]. After sequence confirmation, plasmids were transformed into *A. tumefaciens* GV2260 and gene silencing was performed as described previously [64].

### Screening of effector-triggered immunity-specific genes using programmed cell death assays

*A. tumefaciens* GV2260 strains carrying pBTEX:*Pto* or pBTEX:*avrPto* were grown overnight on solid LB medium (rifampicin and kanamycin). The following day the strains were incubated for 5 h in induction medium (0.05 M MES, 0.5% D-glucose, 0.0272% NaH_2_PO4, pH 5.6) supplemented with 20× AB salts (2% NH_4_Cl, 0.6% MgSO_4_.7H_2_O, 0.3% KCl, 0.005% FeSO_4_.7H_2_O, 0.2% CaCl_2_.2H_2_O) and 200 μM acetosyringone. Induced cultures were washed with 10 mM MES, 10 mM MgCl_2_ and combined in a 1:1 ratio with a final OD_600_ = 0.15 and 200 μM acetosyringone. The resulting suspensions were syringe-infiltrated into leaves of the silenced plants. Scoring was performed visually during several days as follows: PCD = more than 20% of the infiltrated area (within a marked circle) showed cell death; no PCD = less than 20% of the infiltrated area showed cell death. The number of infiltrated areas in each category was then used to calculate the PCD percentage. Plants silenced for *GFP* and an *E. coli* gene-based fragment (*Ec1*, which contains a 56% GC and not a single ≥17 bp-long 100% identical stretch in *N. benthamiana*) were used as negative controls. *MAPKKK*α, *MEK2* and *Prf* were used as positive controls. The gene fragments used for silencing are provided in Additional file 7.

### Measurement of reactive oxygen species production

Discs from young leaves of *Ec1*-, *SlEpk1*- and *NbFls2*-silenced plants were excised with a 4-mm-diameter cork borer. Leaf disks were floated adaxial side up in a 96-well black plate (Greiner Bio-one, Kremsmuenster, Austria) containing 180 μl of water per well, and left at room temperature overnight. The next day, the water was removed, and 100 μl of a solution containing the following was added: 500 nM flg22 (GenScript, Piscataway, NJ USA), luminol at 34 μg/ml, and horseradish peroxidase at 20 μg/ml (Sigma, St Louis, MO, USA) in water. Luminescence was measured using the Synergy HT plate reader (Biotek, Winooski, VT, USA). Four leaf disks per plant were taken, and six plants silenced for each gene were considered in each experiment.

### Disease assays using *Pseudomonas syringae* pv. *tabaci*

*N. benthamiana* (Nb) or Nb-35S:*Pto* VIGS-silenced plants were syringe- or vacuum-infiltrated with 5 × 10^4^ cfu/ml *P. s. tabaci* expressing HopQ1-1, AvrPto or carrying an empty vector. The plants were scored as described in the PCD assay above for the presence of disease-associated cell-death and used for bacterial growth assays. Fisher’s exact test (*P* <0.05) was used to determine significant differences.

### Bacterial population assays

Seven-week-old VIGS-silenced plants were vacuum-infiltrated with a suspension of 5 × 10^4^ cfu/ml *P. s. tabaci* (HopQ1-1) or 6 × 10^4^ cfu/ml *P. s. tabaci* (AvrPto) in 10 mM MgCl_2_ and 0.002% Silwet L-77. To measure bacterial populations, three 0.43 cm^2^ disks were taken from the oldest expanding leaves and processed twice in a Tissue Lyser (Qiagen, Germantown, MD, USA) for 30 s at 25/s frequency with 0.25 ml of 10 mM MgCl_2_. The volume was adjusted to 1 ml and serial dilutions were plated on solid LB medium with antibiotics. In each experiment, six biological replicates per construct were used. A Student’s *t*-test (*P* <0.05) was used to determine significant differences.

### *Agrobacterium*-mediated transient assays

ETI elicitors were transiently expressed in VIGS-silenced *N. benthamiana* plants to induce PCD. Pto, AvrPto_1–387_, Rx2/CP, RPP13/ATR13Δ41 and Gpa2/RBP-1 were expressed under the 35S promoter (pBTEX), SlMAPKKKα full-length, SlMAPKKKα KD^-^, MEK2^DD^ and MEK2 WT were expressed using an estradiol-inducible system (pER8), and Bax was expressed using a dexamethasone-inducible system (pTA7002). Expression was induced 48 hai with 2 μM estradiol or 10 μM dexamethasone mixed with 0.02% Tween 20.

### *SlEpk1*-silencing efficiency

Total RNA isolation, DNAse treatment, cDNA synthesis and qRT-PCR were performed as described above, using *N. benthamiana* leaf tissue from *Ec1*- and *Epk1*-silenced plants. The genes chosen for the analysis are shown in Additional file 10. Primers used for each gene analyzed were: Nb00051202g0009.1 F: 5′-TGTTGGGTCAAATGATTCTCACA-3′, R: 5′- GCTCAACCCATTAGAAACTCTGA-3′; Nb00020954g0005.1 F: 5′-TGTCGGGTCAAATGATTCTC-3′, R: 5′-CAACCCGTTAGAAACTCTCC-3′; Nb00014536g0001.1 F: 5′-AGGCTACCTAATGATGATGAAA-3′, R: 5′-GGTGGAGTTGAGACAATAGAG-3′; Nb00003176g0019.1 F: 5′-ACCATGAACATTATGGACTGT-3′, R: 5′-CAACAGAACCTCCACCATT-3′; Nb00029791g0013.1 F: 5′-AGAGGCTCCAAAGTTCGCA-3′, R: 5′-AACCGAACCTCCACCAAT-3′; Nb00042373g0002.1 F: 5′-ATGATAAGACTACCTGATGATGAC-3′, R: 5′-GGTGGAGTTGAGATAAGAGAATAA-3′. Data were normalized using *NbPP2a* and *NbEF1*α [65]. Cycling conditions during qRT-PCR were 50°C for 2 minutes, 95°C for 10 minutes, and 40 cycles of 95°C for 30 s, 58°C for 30 s and 72°C for 30 s. A pairwise Student’s *t*-test (*P* <0.01) was used to determine significance differences.

### Gene Ontology term analysis

Genes with expression levels ≥3 RPKM in at least one treatment, >2-fold induction and *P* <0.05 were used for the GO term analysis using the GO::Term Finder module [66].

### Phylogenetic analysis

The SeaView program [67] was used to perform the phylogeny analysis with the default parameters for the GTR model (nucleotides) and the JTT model (proteins). PhyML with one hundred bootstraps was used for each analysis. The tree figure was created using FigTree [68].

### Bibliographic search associated with effector-triggered and pattern-triggered immunity-induced genes

SwissProt and TrEMBL protein databases [69], and EMBL-EBI plant EST and STD nucleotides databases [70] were downloaded and filtered using Ruby custom scripts to obtain plant sequences with PUBMED publications [71]. These sequences were compared with the tomato gene models (ITAG2.3) using BLAST [72] to find putative orthologs to the tomato genes. Custom Ruby and BioRuby [73] scripts, available upon request, were used to filter the bibliography to obtain publications related to plant immunity associated with these genes.

### Data access

The RNA-Seq data are available from the NCBI (accessions SRP043126 and SRP043127) and the Tomato Functional Genomics Database [60] (accessions D010 and D011).

## Additional files

Additional file 1: Figure S1. Transcriptome analysis of Pto/Prf-mediated ETI in tomato. **(A)** Summary of the experimental strategy. RG-PtoR (resistant), RG-*prf3* and RG-*prf19* (susceptible) tomato plants were vacuum-infiltrated with *P. syringae* pv. *tomato* DC3000. Leaf tissue was collected after 4 and 6 hai and used to develop RNA-Seq libraries. **(B)** Comparison of disease symptoms between treatments. Photographs were taken 3 dai. **(C)** Total number of genes differentially expressed during ETI (calculated as the ratio between the expression in RG-PtoR and RG-*prf3* or RG-*prf19* plants). The number of genes in each category is shown. **(D)** Percentage and number (in parentheses) of induced genes in each category (ETI, PTI, or both). The comparison was performed using previously published data for PTI-induced genes [13]. A ≥2-fold difference and *P* <0.05 were used as cutoff. RG, Rio Grande; hai, hours after infiltration; dai, days after infiltration. Additional file 2: Figure S2. Populations of DC3000 mutant strains in tomato leaves 6 hai. *Pst* DC3000 Δ*fliC*, *Pst* DC3000 Δ*avrPto* Δ*avrPtoB* and *Pst* DC3000 Δ*avrPto* Δ*avrPtoB* Δ*fliC* populations were measured in leaves. Leaves of tomato RG-PtoR plants were infiltrated with 5 × 10^6^ cfu/ml DC3000 mutant strains and sampled to measure bacterial populations at 6 hai. Bars represent the mean of four plants per strain with their corresponding standard error. Different letters indicate significant differences at 6 h using Tukey’s HSD test (*P* <0.05). Additional file 3: Table S1. ETI-, PTI- and common-induced gene expression data. Reference column shows information about genes previously described to participate in plant immunity (see Materials and methods for detailed information). Additional file 4: Figure S3. Distribution of induced genes in each category of plant immune response (ETI, PTI or both). **(A)** Analysis using maximum RPKM, **(B)** average RPKM and **(C)** fold change. A ≥2-fold difference and *P* <0.05 were used as cutoff. Additional file 5: Table S2. GO term analysis of ETI-, PTI- and common induced genes. Terms are grouped based on process (P), component (C) and function (F). Additional file 6: Figure S4. Summary of transcription factors (TFs) whose transcript abundance is increased during ETI, PTI or both. **(A)** Percentage and number (in parentheses) of induced TF genes present in each category. **(B)** Number of genes in each TF family induced in ETI, PTI or both. A ≥2-fold difference and *P* <0.05 were used as cutoff. Additional file 7: Table S5. Nucleotide sequences of fragments used for VIGS. Additional file 8: Figure S5. Identification of *SlEpk1* in a screen using Pto/AvrPto in *N. benthamiana.* **(A)** Leaves of *N. benthamiana* plants silenced for the genes shown were syringe-infiltrated with *Agrobacterium* carrying Pto/AvrPto to elicit programmed cell death (PCD). The degree of PCD was monitored visually (see Materials and methods). **(B)** Production of flg22-induced reactive oxygen species (ROS). Leaf disks from silenced plants were treated with 500 nM flg22 and ROS production was measured. The level of ROS at different time points is shown as relative light units (R.L.U.), and the names of the genes silenced are indicated. Six silenced plants were tested for each gene, and the average R.L.U. with standard error is shown. **(C)** Percentage of silencing in *Ec1-* and *SlEpk1-*silenced plants using qRT-PCR. Silencing efficiency is shown as relative expression compared with the *Ec1* control. *NbPP2a* was used as the reference gene and similar results were obtained using *NbEF1*α. Asterisks indicate significant differences compared with *Ec1*-silenced plants using a Student’s *t*-test (*P* <0.05). A, NbS00020954g0005.1; B, NbS00051202g0009.1; C, NbS00029791g0013.1; D, NbS00003176g0019.1; E, NbS00042373g0002.1; F, NbS00014536g0001.1; G, *NbEF1*α. Additional file 9: Figure S6. Phylogenetic analysis of the GmPK6/AtMRK1-like protein kinase family using amino acid sequences. Green, black and red font and lines indicate *Arabidopsis*, *N. benthamiana* and tomato proteins, respectively. SlEpk1 is labeled next to the corresponding accession number and marked with a red star. Details of the SlEpk1 clade are shown in the upper-right corner. The PhyML method with a bootstrap of 100 replicates was used for the analysis (SeaView software [67]). Additional file 10: Table S3. Analysis of *SlEpk1* silencing in *N. benthamiana*. The length of perfect-match stretches of ≥17 nucleotides is shown. Possible target genes in *N. benthamiana* are shown in bold font. N.C., not considered due to low/no expression or short sequence encoding a likely non-functional protein. Additional file 11: Figure S7. Phylogenetic analysis of GmPK6/AtMRK1-like kinase nucleotide sequences in *Nicotiana benthamiana*. Accession numbers in black show the possible targets of the tomato VIGS construct in *N. benthamiana* (see Additional file 10: Table S3). Tomato *Epk1* was added to the analysis (red) and clades A and B are marked with a black square. The PhyML method with a bootstrap of 100 replicates was used for the analysis (SeaView software, [67]). Additional file 12: Table S4. Bacterial strains used in this study. Additional file 13: Table S6. Summary of the sequencing data for each of the libraries generated in this work.

## Abbreviations

bp: base pair
cfu: colony-forming unit
ETI: effector-triggered immunity
GFP: green fluorescent protein
GO: Gene Ontology
hai: hours after inoculation
LB: Luria-Bertani
MAMP: microbe-associated molecular pattern
MAPK: mitogen-activated protein kinase
Nb: *Nicotiana benthamiana*
NB-LRR: nucleotide binding-leucine-rich repeat
PCD: programmed cell death
*Pst*: *Pseudomonas syringae* pv. *tomato*
PTI: pattern-triggered immunity
qRT-PCR: quantitative reverse transcription-polymerase chain reaction
RG: Rio Grande
ROS: reactive oxygen species
RPKM: reads per kilobase of exon model per million mapped reads
VIGS: virus-induced gene silencing
