# Supplementary figures and images for: Transcriptomic analysis reveals tomato genes whose expression is induced specifically during effector-triggered immunity and identifies the Epk1 protein kinase which is required for the host response to three bacterial effector proteins
Source: Genome Biol. 2014 Oct 17;15(10):492. doi: 10.1186/s13059-014-0492-1 (PMC4223163; doi:10.1186/s13059-014-0492-1)

**A**

| Plant            | Strain | Concentration          | Time points |
|------------------|--------|------------------------|-------------|
| RG-PtoR          | DC3000 | $2 \times 10^7$ cfu/mL | 4 and 6 h   |
| RG- <i>prf3</i>  |        |                        |             |
| RG- <i>prf19</i> |        |                        |             |

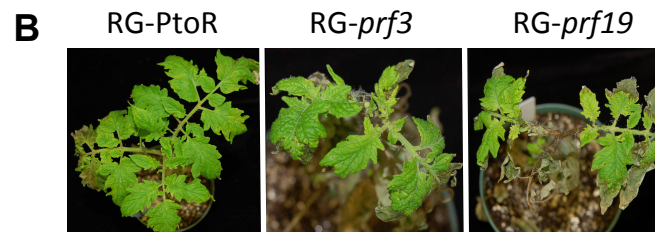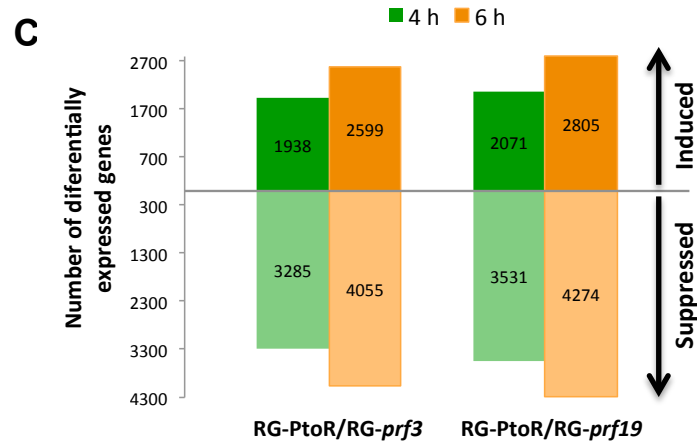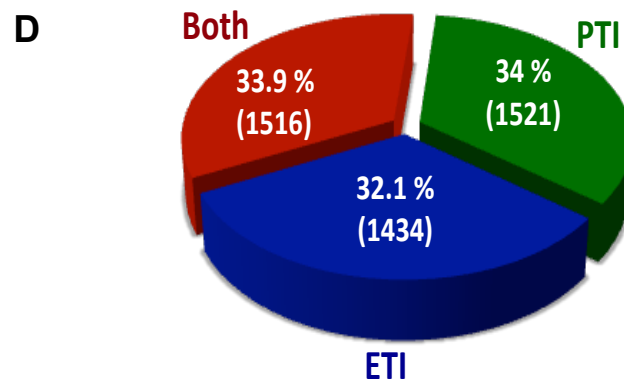

Supplement: Additional file 1: Figure S1. — Transcriptome analysis of Pto/Prf-mediated ETI in tomato. (A) Summary of the experimental strategy. RG-PtoR (resistant), RG-prf3 and RG-prf19 (susceptible) tomato plants were vacuum-infiltrated with P. syringae pv. tomato DC3000. Leaf tissue was collected after 4 and 6 hai and used to develop RNA-Seq libraries. (B) Comparison of disease symptoms between treatments. Photographs were taken 3 dai. (C) Total number of genes differentially expressed during ETI (calculated as the ratio between the expression in RG-PtoR and RG-prf3 or RG-prf19 plants). The number of genes in each category is shown. (D) Percentage and number (in parentheses) of induced genes in each category (ETI, PTI, or both). The comparison was performed using previously published data for PTI-induced genes [13]. A ≥2-fold difference and P <0.05 were used as cutoff. RG, Rio Grande; hai, hours after infiltration; dai, days after infiltration. [file 13059_2014_492_MOESM1_ESM.pdf]

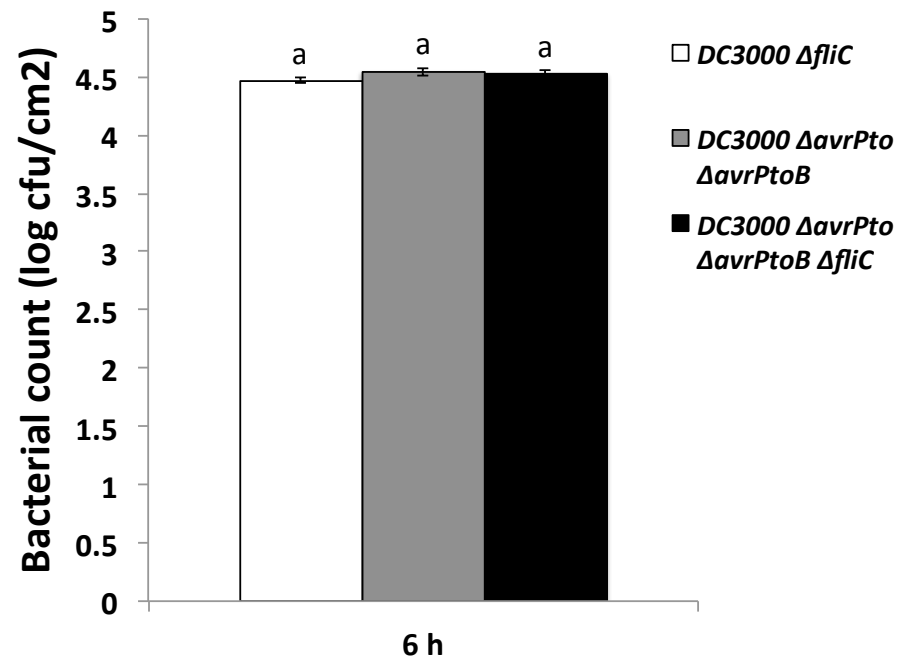

Supplement: Additional file 2: Figure S2. — Populations of DC3000 mutant strains in tomato leaves 6 hai. Pst DC3000 ΔfliC, Pst DC3000 ΔavrPto ΔavrPtoB and Pst DC3000 ΔavrPto ΔavrPtoB ΔfliC populations were measured in leaves. Leaves of tomato RG-PtoR plants were infiltrated with 5 × 10^6^ cfu/ml DC3000 mutant strains and sampled to measure bacterial populations at 6 hai. Bars represent the mean of four plants per strain with their corresponding standard error. Different letters indicate significant differences at 6 h using Tukey’s HSD test (P <0.05). [file 13059_2014_492_MOESM2_ESM.pdf]

**A**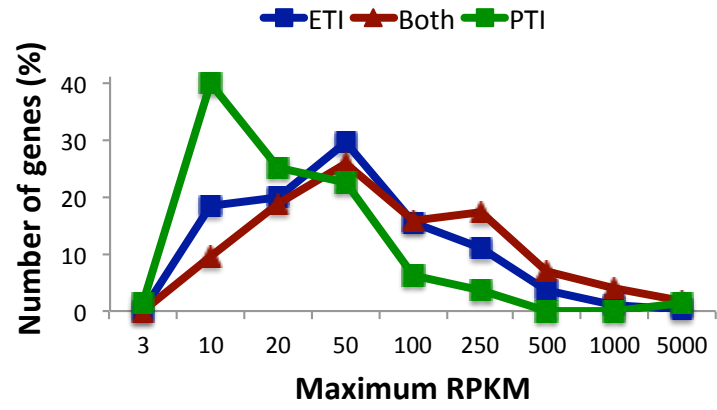**B**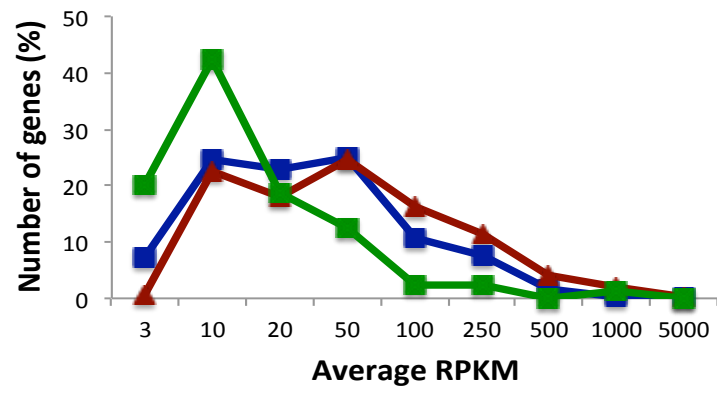**C**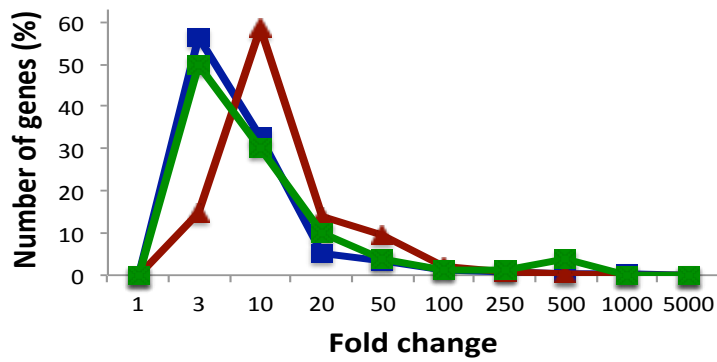

Supplement: Additional file 4: Figure S3. — Distribution of induced genes in each category of plant immune response (ETI, PTI or both). (A) Analysis using maximum RPKM, (B) average RPKM and (C) fold change. A ≥2-fold difference and P <0.05 were used as cutoff. [file 13059_2014_492_MOESM4_ESM.pdf]

A

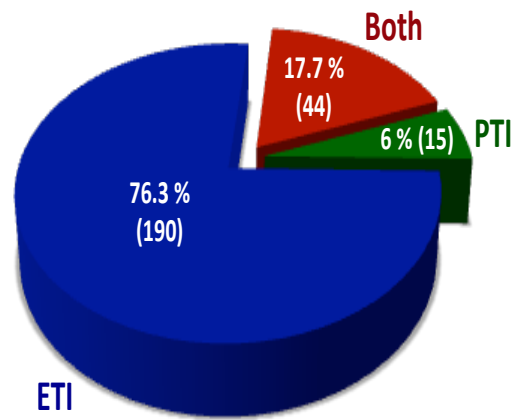

B

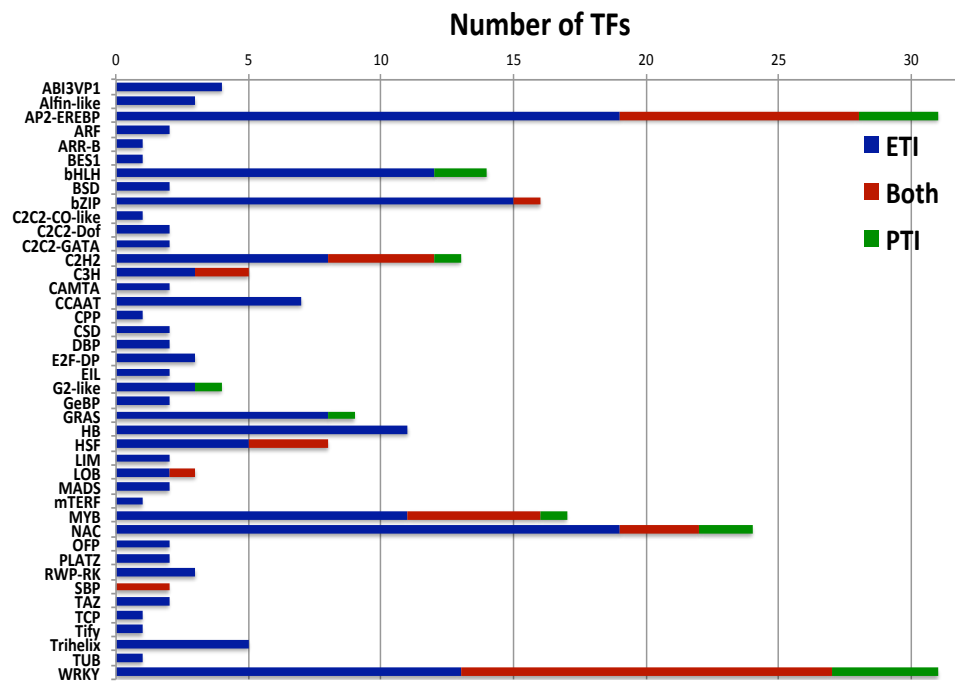

Supplement: Additional file 6: Figure S4. — Summary of transcription factors (TFs) whose transcript abundance is increased during ETI, PTI or both. (A) Percentage and number (in parentheses) of induced TF genes present in each category. (B) Number of genes in each TF family induced in ETI, PTI or both. A ≥2-fold difference and P <0.05 were used as cutoff. [file 13059_2014_492_MOESM6_ESM.pdf]

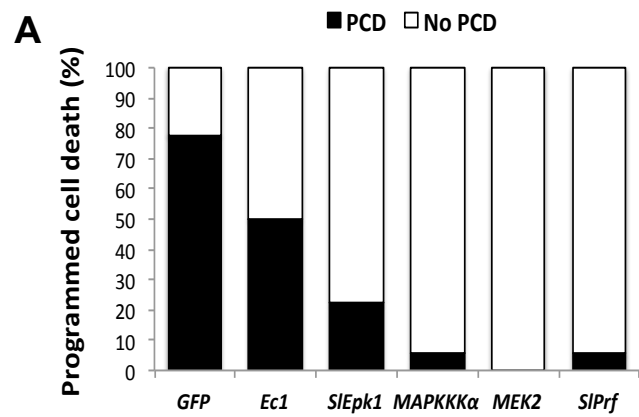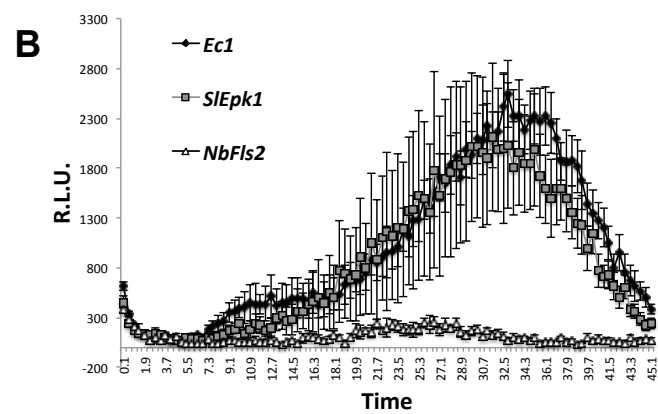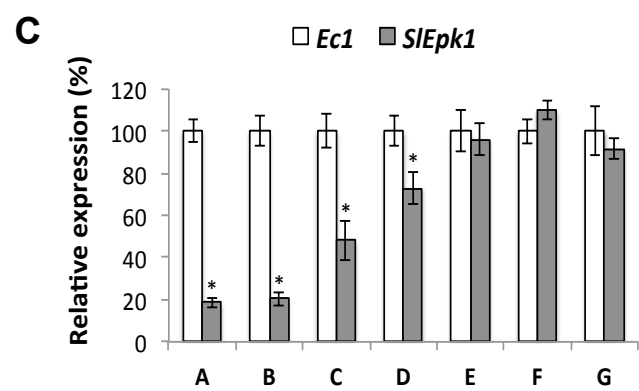

Supplement: Additional file 8: Figure S5. — Identification of SlEpk1 in a screen using Pto/AvrPto in N. benthamiana. (A) Leaves of N. benthamiana plants silenced for the genes shown were syringe-infiltrated with Agrobacterium carrying Pto/AvrPto to elicit programmed cell death (PCD). The degree of PCD was monitored visually (see Materials and methods). (B) Production of flg22-induced reactive oxygen species (ROS). Leaf disks from silenced plants were treated with 500 nM flg22 and ROS production was measured. The level of ROS at different time points is shown as relative light units (R.L.U.), and the names of the genes silenced are indicated. Six silenced plants were tested for each gene, and the average R.L.U. with standard error is shown. (C) Percentage of silencing in Ec1- and SlEpk1-silenced plants using qRT-PCR. Silencing efficiency is shown as relative expression compared with the Ec1 control. NbPP2a was used as the reference gene and similar results were obtained using NbEF1α. Asterisks indicate significant differences compared with Ec1-silenced plants using a Student’s t-test (P <0.05). A, NbS00020954g0005.1; B, NbS00051202g0009.1; C, NbS00029791g0013.1; D, NbS00003176g0019.1; E, NbS00042373g0002.1; F, NbS00014536g0001.1; G, NbEF1α. [file 13059_2014_492_MOESM8_ESM.pdf]

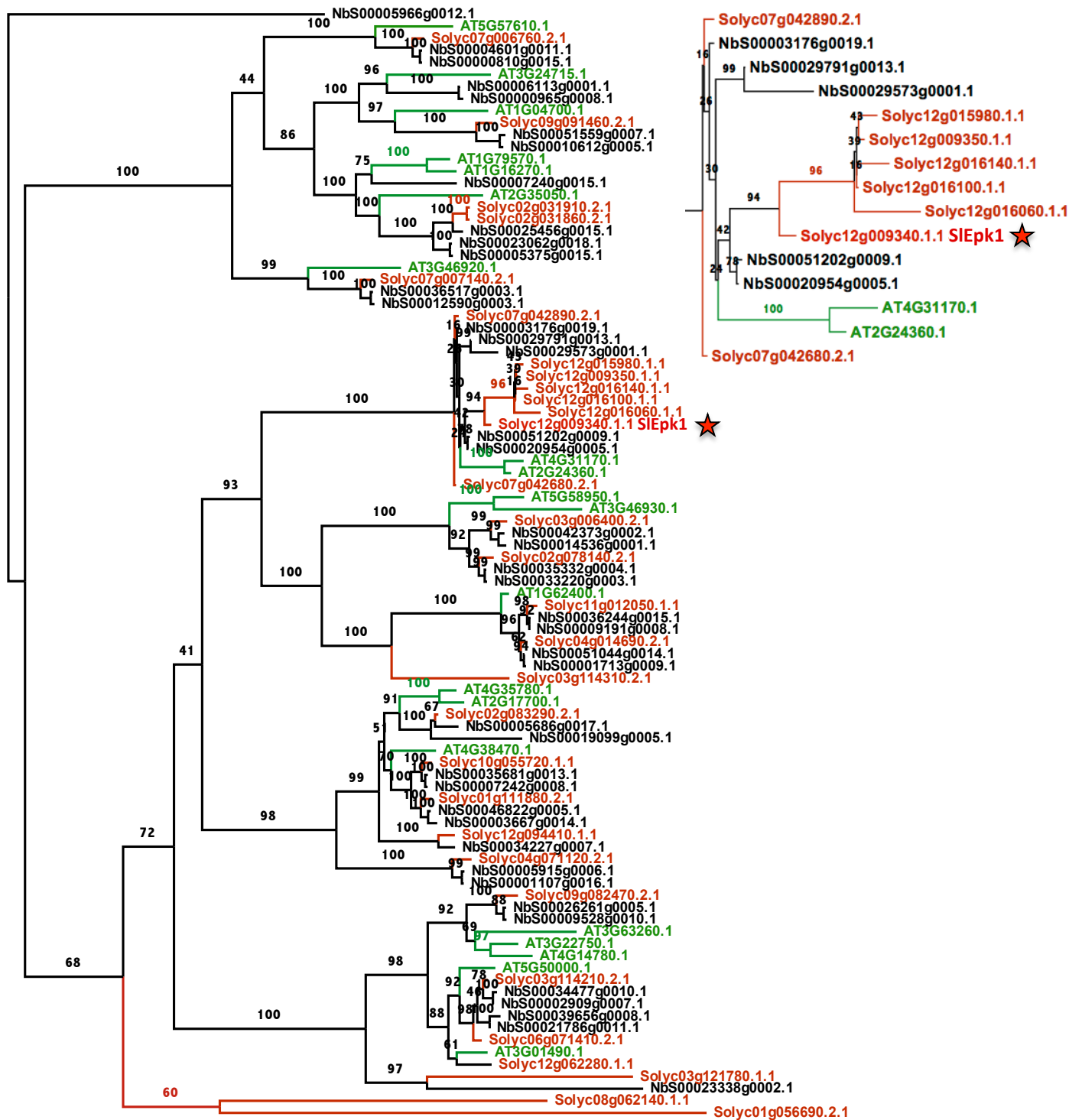

0.6

Supplement: Additional file 9: Figure S6. — Phylogenetic analysis of the GmPK6/AtMRK1-like protein kinase family using amino acid sequences. Green, black and red font and lines indicate Arabidopsis, N. benthamiana and tomato proteins, respectively. SlEpk1 is labeled next to the corresponding accession number and marked with a red star. Details of the SlEpk1 clade are shown in the upper-right corner. The PhyML method with a bootstrap of 100 replicates was used for the analysis (SeaView software [67]). [file 13059_2014_492_MOESM9_ESM.pdf]

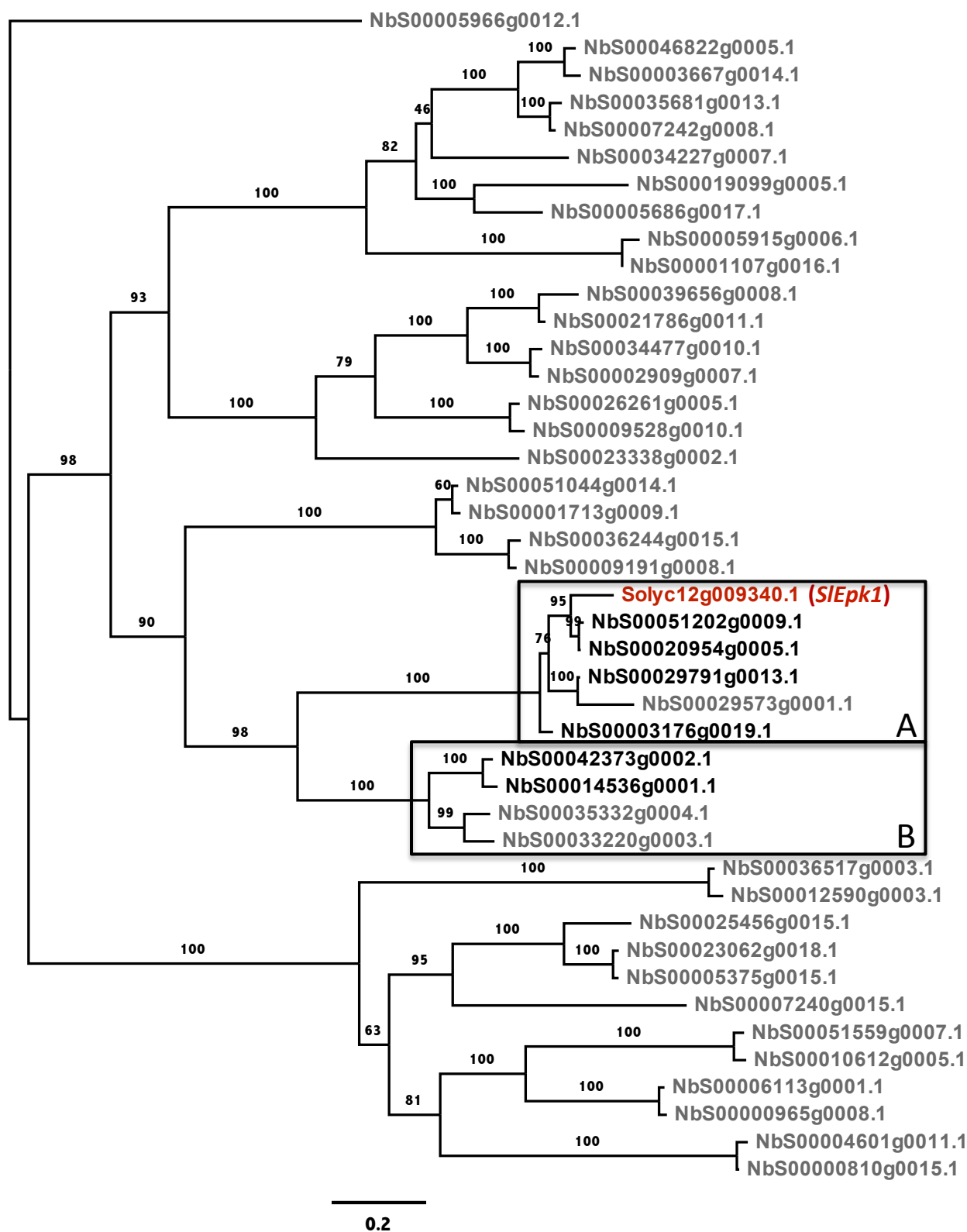

Supplement: Additional file 11: Figure S7. — Phylogenetic analysis of GmPK6/AtMRK1-like kinase nucleotide sequences in Nicotiana benthamiana. Accession numbers in black show the possible targets of the tomato VIGS construct in N. benthamiana (see Additional file 10: Table S3). Tomato Epk1 was added to the analysis (red) and clades A and B are marked with a black square. The PhyML method with a bootstrap of 100 replicates was used for the analysis (SeaView software, [67]). [file 13059_2014_492_MOESM11_ESM.pdf]
