# Supplementary material for: Transcriptomic analysis reveals tomato genes whose expression is induced specifically during effector-triggered immunity and identifies the Epk1 protein kinase which is required for the host response to three bacterial effector proteins
Source: Genome Biol. 2014 Oct 17;15(10):492. doi: 10.1186/s13059-014-0492-1 (PMC4223163; doi:10.1186/s13059-014-0492-1)
Supplement: Additional file 7: Table S5. — Nucleotide sequences of fragments used for VIGS. [file 13059_2014_492_MOESM7_ESM.pdf]

Additional file 12: Table S5. Nucleotide sequences of fragments used for VIGS

Gene	DNA sequence or reference
Ec1 (Escherichia coli fragment*)*	CGGCGTGATTGCGCAAAGCTATCATCAGTCTGAGAAATCGGCCTCCGAGTTCGATGCCA<br>TTGTTGCGCAAACGGAGCAGTTCCTTGCCGACAATGGTCGTCGCCCGCGCATTCTGATC<br>GCTAAGATGGGCCAGGATGGACACGATCGCGGCGCGAAAGTGATCGCCAGCGCCTATTC<br>CGATCTCGGTTTCGACGTAGATTTAAGCCCGATGTTCTCTACACCTGAAGAGATCGCCC<br>GCCTGGCCGTAGAAAACGACGTTCACGTAGTGGGCGCATCCTCACTGGCTGCCGGTCAT<br>AAAACGCTGATCCCGGAACTGGTCGAAGCGCTGAAAAAATGGGGACGCGAAGATATCT<br>GCGTGGTCGCGGGTGGCGTCATTCCGCCGCAGGATTACGCCTTCCTGCAAGAGCGCGGC<br>GTGGCGGCGATTTATGGTCCAGGTACACCTATGCTCGACAGTGTGCGCGACGTACTGAA<br>TCTGATAAGCCAGCATCATGATTAATGAAGCCACGCTGGCAGAAAGTATTCG
GFP	CCTCGGCCGAATTCAGTAAAGGAGAAGAACTTTTCACTGGAGTTGTCCCAATTCTTGTT<br>GAATTAGATGGTGATGTTAATGGGCACAAATTTTCTGTCAGTGGAGAGGGTGAAGGTG<br>ATGCAACATACGGAAAACTTACCCTTAAATTTATTTGCACTACTGGAAAACTACCTGTT<br>CCATGGCCAACACTTGTCACTACTTTCTCTTATGGTGTTCAATGCTTTTCAAGATACCC<br>AGATCATATGAAGCGGCACGACTTCTTCAAGAGCGCCATGCCTGAGGGATACGTGCAGG<br>AGAGGACCATCTTCTTCAAGGACGACGGGAACTACAAGACACGTGCTGAAGTCAAGTT<br>TGAGGGAGACACCCTCGTCA
NbMAPKKKα	CAAGGTCTACAAAAGTTACTCATGTTGGTGTAACCAACGAAGCATATCCTCGTTCCTTT<br>GATGGAAACCGGACGCCGCCAGTGTTGGATTCCGGTGGAAGGAATATTTCTCCAACTAA<br>AGGAAACTATGCAAGCCATCCAGTGATCACCATCTCTAGACCATTGAGCTGCCCAAGGG<br>AAATTGTAAAAACCATAACATCTTTACCAGTATCCCCCACTTCTAGCCCATTAAGACAG<br>TATGAACCTGCACGTAGGAGCTGTTATCTTTCTCTTCCCCACCCAGCTTATGGTATTGG<br>GGGTCATAGTGGATATGATGAGAATGATTACTTAATGTTTCAAGCCAGACCCACCACA<br>AGAAACACACTAGAACCATGGCTTGAAATCCCTCAATTTAAAGTTCAGACACCGTCTAG<br>ATCGCCAAAACTAAGACCAATTCTTTAGTACCTCGGCCGCGACCACCGTACCCAGCTTT<br>CTTGTACAAAGTGGTGATATCGTCGACCGCCGATGAACGGTTAAGTTTCCATTGATACT<br>CGAAAGATGTCAGCACCAGCTAGCACAACACAGCCCATAGGGTCAACTACCTCAACTAC<br>CACAAAAACTGCAGGCGGCAACTCCNTGCCACAGCTTCAGGCCTGTTCACTATCCCGGA<br>TGGGGGATTTCTTTA
NtMEK2	[1]
SlPrf	TGTCATCTCAATCGTTGGCATGCCAGGATTGGGCAAGACTACACTAGCAAAGAAGATT<br>TACAATGATCCAGAAGTCACCTCTCGCTTCGATGTCCATGCTCAATGTGTTGTGACTCA<br>ATTATATTCATGGAGAGAGTTGTTGCTCACCATTTTGAATGATGTGCTTGAGCCTTCTG<br>ATCGCAATGAAAAAGAAGATGGAGAAATAGCTGATGAGCTACGCCGATTTTTGTTGAC<br>CAAGAGATTCTTGATTCTCATTGATGATGTGTGGGAC
NbSAG101	TGCTAGGTCTTTTGGGGTTCTTCAACTTACTCCAAAGGCCAAGAATATGAGTCAGGATT<br>CCTTGTTTAGTAGTGGCCAAGAATTGGCAAAGTTGGTGGTGAGCTCAGATCTACTGCA<br>TGATTCTTGGGCTAGAAATTGTGATCTTCTTAATCATGCTTATTTGGATAATCCAACTA<br>ACCCAGCTCCAATTGTGTTCAAAGTTTATTACCCATATTATACAAATGGTGCTATTGTT<br>GCTTTTGTATCCTCCCCTACCTGTAGTATTCATCATCTTCAGAAAGAAATGGTCTCTTC<br>AGAAGATCTTAAAGGTTCCCAAGTTGATTTTGA
SlEpk1	ACCGGTCATACACCCAGAAAGTTGATGTTTATAGTTTTGGCATTGTTCTGTGGGAGCTC<br>ATAACTGGGTTGCTTCCTTTCCAGAACATGACTGCTGTGCAGGCTGCTTTTGCTGTTGT<br>CAACAAAGGCGTCCGTCCAACAATCCCCATTGATTGTTTGCCTGTCTTATCTGATATCA<br>TGACCTGCTGCTGGGATTCTGACCCCAATAATAGGCCAACTTTCTCTCAGGTTGTCAAG<br>ATGCTTGAGGCAGCTGAGACAGAA

1. Ekengren SK, Liu Y, Schiff M, Dinesh-Kumar SP, Martin GB: **Two MAPK cascades, NPR1, and TGA transcription factors play a role in Pto-mediated disease resistance in tomato.** *Plant J* 2003, 36:905-917.
