# Supplementary material for: Transcriptomic analysis reveals tomato genes whose expression is induced specifically during effector-triggered immunity and identifies the Epk1 protein kinase which is required for the host response to three bacterial effector proteins
Source: Genome Biol. 2014 Oct 17;15(10):492. doi: 10.1186/s13059-014-0492-1 (PMC4223163; doi:10.1186/s13059-014-0492-1)
Supplement: Additional file 10: Table S3. — Analysis of SlEpk1 silencing in N. benthamiana. The length of perfect-match stretches of ≥17 nucleotides is shown. Possible target genes in N. benthamiana are shown in bold font. N.C., not considered due to low/no expression or short sequence encoding a likely non-functional protein. [file 13059_2014_492_MOESM10_ESM.pdf]

Additional file 10: Table S3. Analysis of *SlEpk1* silencing in *N. benthamiana*. The length of perfect-match stretches of ≥17 nucleotides is shown. Possible target genes in *N. benthamiana* are shown in bold font. N.C., not considered due to low/no expression or short sequence encoding a likely non-functional protein.

Gene	Nb-35S:Pto DC3000 *ΔhopQ1-1* expression (RPKM)	Length (bp) of perfect match stretches ≥ 17 bp
**NbS00051202g0009.1**	**79.83**	**43, 28, 29**
**NbS00020954g0005.1**	**83.95**	**32, 28, 29**
NbC23305057g0001.1	66.97	N.C.
**NbS00014536g0001.1**	**4.71**	**26**
NbS00023433g0102.1	41.29	N.C.
**NbS00003176g0019.1**	**53.86**	**20, 26**
NbS00029573g0002.1	67.68	N.C.
**NbS00029791g0013.1**	**51.34**	**20, 23**
**NbS00042373g0002.1**	**5.33**	**23**
NbS00000863g0005.1	0	N.C.
NbS00014032g0110.1	0.72	N.C.
NbS00039241g0001.1	0	N.C.
