# Supplementary material for: Transcriptomic analysis reveals tomato genes whose expression is induced specifically during effector-triggered immunity and identifies the Epk1 protein kinase which is required for the host response to three bacterial effector proteins
Source: Genome Biol. 2014 Oct 17;15(10):492. doi: 10.1186/s13059-014-0492-1 (PMC4223163; doi:10.1186/s13059-014-0492-1)
Supplement: Additional file 12: Table S4. — Bacterial strains used in this study. [file 13059_2014_492_MOESM12_ESM.pdf]

Additional file 11: Table S4. Bacterial strains used in this study.

Strain	Characteristics	Reference
Agrobacterium tumefaciens GV2260	Disarmed Ti plasmid; Rif^R	[1]
Escherichia coli DH5α	F– Φ80*lac*ZΔM15 Δ(*lac*ZYA-*arg*F) U169 *rec*A1 *end*A1 *hsd*R17 (rK–, mK+) *pho*A *sup*E44 λ– *thi*-1 *gyr*A96 *rel*A1	Invitrogen
Pseudomonas syringae pv. *tomato* (*Pst*) DC3000	Wild type; Rif^R	[2]
Pst DC3000 Δ*hopQ1-1*	Δ*hopQ1-1;* Rif^R	[3]
Pst DC3000 Δ*hopQ1-1* Δ*fliC*	Δ*hopQ1-1* Δ*fliC;* Rif^R	[4]
Pst DC3000 Δ*hopQ1-1* Δ*avrPto* Δ*avrPtoB*	Δ*hopQ1-1* Δ*avrPto* Δ*avrPtoB;* Rif^R	[4]
Pst DC3000 Δ*hopQ1-1* Δ*fliC* Δ*avrPto* Δ*avrPtoB*	Δ*hopQ1-1* Δ*fliC* Δ*avrPto* Δ*avrPtoB;* Rif^R	[4]
Pseudomonas syringae pv. *tabaci* (*P. s. tabaci*)	Wild type; Rif^R	[5]
P. s. tabaci HopQ1-1	pCPP5372::*hopQ1-1*; Rif^R Gen^R	[4]
P. s. tabaci AvrPto	pDSK519::*avrPto*; Rif^R Kan^R	[5]
P. s. tabaci AvrPto-I96A	pDSK519::*avrPto-I96A*; Rif^R Kan^R	[6]
P. s. tabaci empty vector	pDSK519; Rif^R Kan^R	[6]

Rif: rifampicin; Gen: gentamycin; Kan: Kanamycin.

1. McBride K, Summerfelt K: **Improved binary vectors for *Agrobacterium*-mediated plant transformation.** *Plant Molecular Biology* 1990, **14:**269-276.

2. Cuppels DA: **Generation and characterization of Tn5 insertion mutations in *Pseudomonas syringae* pv. *tomato*.** *Applied and Environmental Microbiology* 1986, **51:**323-327.

3. Wei CF, Kvitko BH, Shimizu R, Crabill E, Alfano JR, Lin NC, Martin GB, Huang HC, Collmer A: **A *Pseudomonas syringae* pv. *tomato* DC3000 mutant

lacking the type III effector HopQ1-1 is able to cause disease in the model plant *Nicotiana benthamiana*. *Plant J* 2007, **51:**32-46.

4. Kvitko BH, Park DH, Velasquez AC, Wei CF, Russell AB, Martin GB, Schneider DJ, Collmer A: **Deletions in the repertoire of *Pseudomonas syringae* pv. *tomato* DC3000 type III secretion effector genes reveal functional overlap among effectors.** *PLoS Pathog* 2009, **5:**e1000388.

5. Thilmony RT, Chen Z, Bressan RA, Martin GB: **Expression of the tomato *Pto* gene in tobacco enhances resistance to *Pseudomonas syringae* pv. *tabaci* expressing *avrPto*.** *Plant Cell* 1995, **7:**1529-1536.

6. Yeam I, Nguyen HP, Martin GB: **Phosphorylation of the *Pseudomonas syringae* effector AvrPto is required for FLS2/BAK1-independent virulence activity and recognition by tobacco.** *Plant J* 2010, **61:**16-24.
