# Supplementary material for: Transcriptomic analysis reveals tomato genes whose expression is induced specifically during effector-triggered immunity and identifies the Epk1 protein kinase which is required for the host response to three bacterial effector proteins
Source: Genome Biol. 2014 Oct 17;15(10):492. doi: 10.1186/s13059-014-0492-1 (PMC4223163; doi:10.1186/s13059-014-0492-1)
Supplement: Additional file 13: Table S6. — Summary of the sequencing data for each of the libraries generated in this work. [file 13059_2014_492_MOESM13_ESM.pdf]

Additional file 13: Table S6: Summary of the sequencing data for each of the libraries generated in this work

Treatment	Replicate	Total reads	rRNA reads	% rRNA	Clean reads	Mapped reads	% Mapped
**PtoR-DC3000**	**1**	9,994,651	36,242	0.3	9,958,409	8,993,856	90.31
	**2**	16,861,386	103,085	0.6	16,758,301	15,771,011	94.11
	**3**	13,054,963	51,972	0.4	13,002,991	12,109,859	93.13
	**Average**	13,303,667	63,766	0.4	13,239,900	12,291,575	92.5
***prf3*-DC3000**	**1**	14,467,697	102,886	0.7	14,364,811	13,026,233	90.68
	**2**	20,262,032	473,515	2.3	19,788,517	18,621,095	94.1
	**3**	12,560,590	58,207	0.5	12,502,383	11,609,398	92.86
	**Average**	15,763,440	211,536	1.2	15,551,904	14,418,909	92.5
***prf19*-DC3000**	**1**	15,511,557	91,430	0.6	15,420,127	13,875,215	89.98
	**2**	20,223,358	503,397	2.5	19,719,961	18,360,424	93.11
	**3**	11,194,497	66,738	0.6	11,127,759	10,362,546	93.12
	**Average**	15,643,137	220,522	1.2	15,422,616	14,199,395	92.1
**Total average**		14,903,415	165,275	0.944	14,738,140	13,636,626	92.38
**PtoR-DC3000 *ΔfliC***	**1**	14,935,433	5,125,505	34.3	9,809,928	9,114,331	92.91
	**2**	12,323,202	3,431,511	27.8	8,891,691	8,347,476	93.88
	**3**	15,199,111	2,657,884	17.4	12,541,227	11,843,170	94.43
	**Average**	14,152,582	3,738,300	26.5	10,414,282	9,768,326	93.74
**PtoR-DC3000 *ΔavrPto ΔavrPtoB***	**1**	15,647,847	8,880,310	56.7	6,767,537	6,250,683	92.36
	**2**	19,905,252	1,428,189	7.1	18,477,063	17,344,002	93.87
	**3**	12,248,832	2,938,586	23.9	9,310,246	8,728,766	93.75
	**Average**	15,933,977	4,415,695	29.23	11,518,282	10,774,484	93.33
**PtoR-DC3000 *ΔfliC ΔavrPto ΔavrPtoB***	**1**	13,620,317	4,802,212	35.2	8,818,105	8,214,410	93.15
	**2**	9,938,524	2,351,212	23.6	7,587,312	7,090,050	93.45
	**3**	12,109,823	4,629,721	38.2	7,480,102	6,983,701	93.36
	**Average**	11,889,555	3,927,715	32.33	7,961,840	7,429,387	93.32
**Total average**		13,992,038	4,027,237	29.36	9,964,801	9,324,065	93.46
